# Coordinated transfer of DNA between Pol θ and Pol δ resets microhomology choice during double-strand break repair

**DOI:** 10.1073/pnas.2513018122

**Published:** 2025-11-19

**Authors:** Yuzhen Li, Mark Returan, Adele T. Guerin, April M. Averill, Dorcas Oladapo, Sylvie Doublié, Richard D. Wood

**Affiliations:** ^a^Department of Epigenetics and Molecular Carcinogenesis, The University of Texas MD Anderson Cancer Center, Houston, TX 77230; ^b^Department of Microbiology and Molecular Genetics, University of Vermont, Burlington, VT 05405

**Keywords:** DNA polymerase, helicase, biochemical reconstitution, TMEJ

## Abstract

DNA polymerase theta (Pol θ)-mediated end joining (TMEJ) initiates DNA double-strand break repair by using short homologies (microhomologies) between single-stranded DNA tails. This repair process is particularly important in cancer cells defective in homologous recombination. The exonuclease function of DNA polymerase delta (Pol δ) has been identified as an essential component for TMEJ, functioning to remove unpaired bases flanking a microhomology (MH). It is not known if the exonuclease removes all unpaired bases at once and how this removal might affect subsequent MH selection. Here, we reconstituted a functional TMEJ repair process using purified human Pol θ and Pol δ. We find that when Pol δ exonuclease excises a nucleotide to generate a new 3′ terminus, Pol θ initiates a new MH search. Pol δ exonuclease removes a single nucleotide at a time, rather than cleaving an unpaired flap, and then transfers the DNA tail to the polymerase site of Pol θ. Specific primer-grasp amino acids in the polymerase domain of Pol θ are important for internal MH anchoring. The helicase-like domain of Pol θ harbors two activities which are recapitulated in reconstituted reactions: strand-capture to bring single-strand tails together and an ATPase activity that alleviates suppression of TMEJ by ssDNA binding protein RPA. This functional reconstitution of TMEJ advances the understanding of how two polymerases with three enzymatic activities orchestrate double-strand break repair.

## Significance

DNA polymerase theta (Pol θ) initiates repair of double-strand breaks by using microhomologies (MH). This process, known as theta (Pol θ)-mediated end joining (TMEJ), is especially important in cancer cells defective in homologous recombination. The exonuclease of Pol δ also functions in TMEJ, but it is unclear how it coordinates with Pol θ. We reconstituted repair with purified human Pol θ (harboring DNA polymerase and ATPase activities) and Pol δ. We find that Pol δ removes a single nucleotide at a time from single-stranded DNA rather than cleaving an unpaired flap flanking a MH, and then transfers the strand to Pol θ, initiating a new MH search. The results show how these activities are coordinated during the initiation of TMEJ.

DNA polymerase θ (Pol θ)-mediated end joining (TMEJ) is a DNA double-strand break repair pathway that works in parallel with homologous recombination (HR) and nonhomologous end joining (NHEJ) (1, 2). TMEJ protects genome integrity by minimizing large-scale genomic damage during double-strand break repair (3). The survival of some HR-deficient cancer cells depends on Pol θ (4, 5). Pol θ is therefore a proposed target for cancer therapy (1–3, 6), and it is crucial to understand the molecular mechanisms underlying Pol θ-mediated repair.

TMEJ repairs DNA double-strand breaks by bridging two single-stranded DNA (ssDNA) 3′ tails formed after nuclease-mediated resection at the break. The key component of TMEJ, Pol θ, is a large protein containing a helicase-like domain (HLD) connected to an A-family polymerase domain (POL) via a mostly disordered central domain (cen) (7, 8). The Pol θ HLD facilitates capture of two ssDNA tails, bringing the termini close together (9, 10). Pol θ POL binds and extends at least one strand from a paired microhomology (MH) to initiate repair synthesis (11, 12). Internal MH are usually located within ~15 nt of a terminus, and so this process generally results in short deletions (1–3, 13, 14).

The microhomologies used by Pol θ are usually embedded within the two resected ssDNA tails rather than being located at the 3′ terminal ends (14, 15). Unpaired 3′ bases flanking the MH must therefore be removed. Although Pol θ POL has an exonuclease-like subdomain, it lacks 3′ to 5′ exonuclease activity (16) or other intrinsic end trimming activity (17). Experiments in cells showed that the nuclease responsible for the removal of unpaired bases is the 3′ to 5′ exonuclease activity of replicative DNA polymerase delta (Pol δ) (11). A physical association between Pol θ and Pol δ was identified via stochastic optical reconstruction microscopy, coimmunoprecipitation, and microscale thermophoresis (11), but it is not known how the exonuclease removes multiple unpaired bases and how action of the exonuclease affects subsequent MH selection. After Pol θ extends from a MH, synthesizing around 6 to 14 nucleotides, the polymerase activity of Pol δ takes over the task of completing synthesis on single-stranded DNA (11, 18).

Moreover, exposed ssDNA tails are quickly bound and protected by Replication Protein A (RPA) (19). This binding to ssDNA prevents spontaneous annealing between microhomologies (20). To allow a MH search, it is proposed that in mammalian cells, a DNA-dependent ATPase function of the Pol θ HLD (7, 21–23) hydrolyzes ATP to counteract RPA binding to DNA (4, 19).

To investigate these steps of TMEJ, we used in vitro reconstitution as a tool to examine the coordination of each component. We found that Pol δ uses its exonuclease activity to trim one nucleotide at a time from a ssDNA 3′-end, transferring the single-stranded DNA to Pol θ for initiation of DNA synthesis. Each exonuclease event resets the MH search by Pol θ.

## Results

### Pol δ and Pol θ Work Together to Perform TMEJ In Vitro.

**Fig 1.**
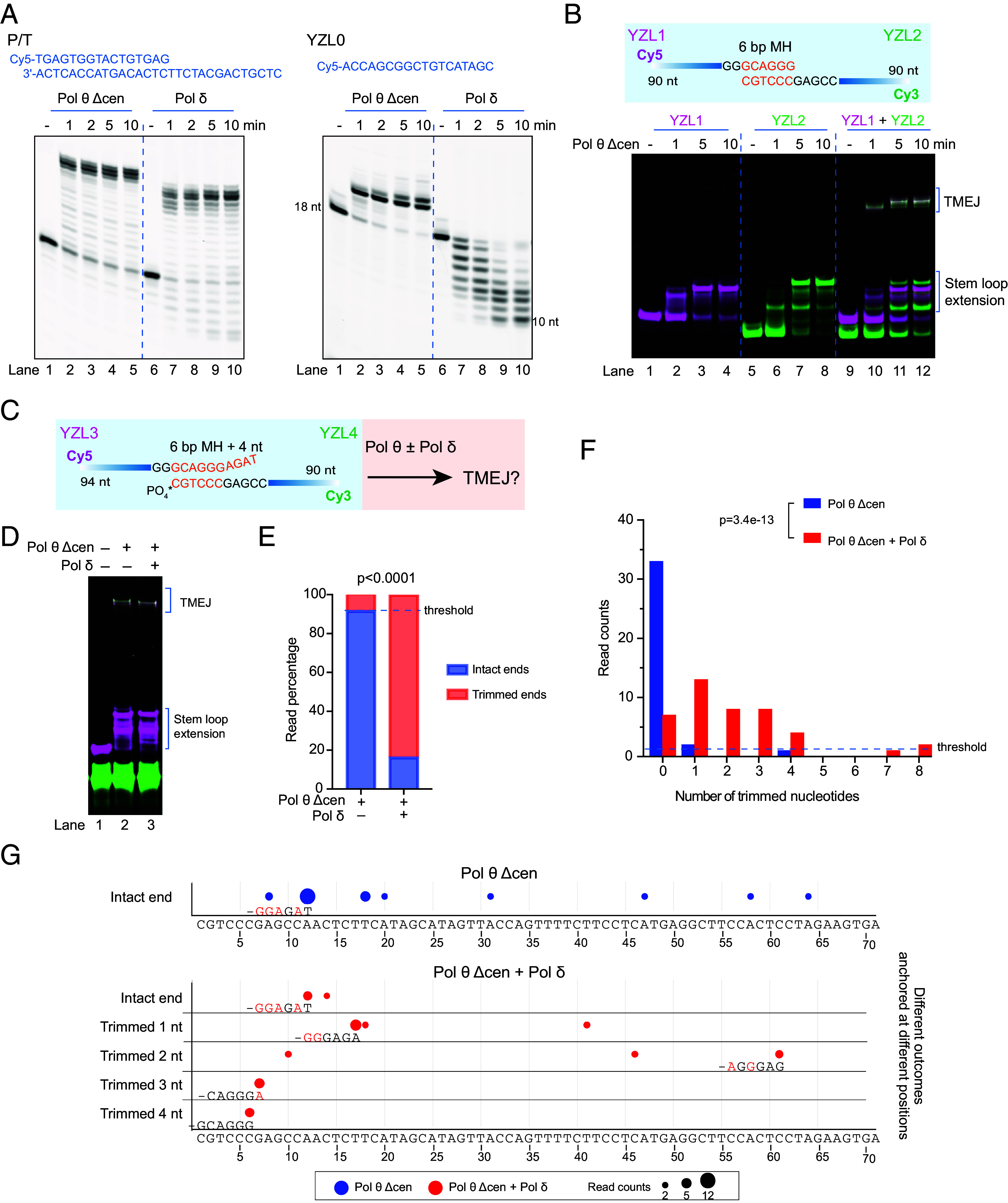
Pol θ and Pol δ work together to perform TMEJ in vitro. (*A*) Activity of Pol θ Δcen and Pol δ on primer-template substrate (P/T) and ssDNA substrate YZL0. 25 nM DNA substrate was incubated with 25 nM Pol θ Δcen or Pol δ in reaction mixtures at 37 °C for the indicated times. Reaction mixtures were separated by electrophoresis on a denaturing 15% polyacrylamide gel. (*B*) TMEJ with Pol θ Δcen and 90-mer oligonucleotides. The schematic shows paired ssDNA substrate containing 5′ fluorescent labels and a terminal 6 bp MH. YZL1 is labeled with Cy5 (magenta) and YZL2 is labeled with Cy3 (green). Reaction mixtures were separated by electrophoresis on a native 10% polyacrylamide gel. 50 nM YZL1 and 50 nM YZL2 were incubated with 100 nM Pol θ Δcen at 37 °C for the indicated times. White bands are TMEJ products. (*C*) Schematic of paired ssDNA substrate (YZL3 and YZL4) containing 5′ fluorescent labels and a 6 bp core MH followed by four unpaired nucleotides. The 3′ end of YZL4 was modified with a phosphate group to block extension and digestion. (*D*) TMEJ reconstitution with Pol θ Δcen, Pol δ, and the ssDNA substrate in (*C*). 12.5 nM YZL3 and 12.5 nM YZL4 were incubated with 50 nM Pol θ Δcen, 50 nM Pol δ and 100 µM dNTPs in reaction buffer at 37 °C for 15 min. Reaction mixtures were separated by electrophoresis on a native 10% polyacrylamide gel. (*E*) Read percentage of TMEJ outcomes from the reactions shown in panel D. Outcomes arising from intact or trimmed ends of YZL3 are compiled from the distributions shown in panel *F*. The statistical significance is labeled with the *P* value derived from two-sided Fisher’s exact test. (*F*) Read counts showing intact or trimmed nucleotide outcomes in reaction products from (*D*). The distributions are statistically different as indicated by the labeled *P* value derived from the Kolmogorov–Smirnov test (KS-test). Sequences represented by only one read were set as a background threshold level for the products with Pol θ Δcen and Pol δ. This is because a few reads in reaction mixtures with Pol θ Δcen only were interpreted as arising from trimmed YZL3. These are likely artifacts ascribable to a low level of incomplete oligonucleotide synthesis, reagent contamination, PCR amplification mutation, or sequencing error. (*G*) MH anchoring positions for the data in panels *E* and *F*. A circle marks each anchoring position of the 3′ end of YZL3 on the YZL4 template sequence. This is the junction where the two ssDNAs are joined by Pol θ. The YZL4 template sequence is shown at the bottom, numbered from the 3′ end. The size of the circle indicates the read count for each outcome. The blue circles are for reactions with Pol θ Δcen only, and the red circles for reactions with both Pol θ Δcen and Pol δ. Outcomes are categorized based on whether they resulted from an intact 3′ end or from a YZL3 3′ end trimmed by one to four nt. The most frequent MH is labeled under the corresponding circle. In the MH, bases in black are matched and bases in red are mismatched. Outcomes with only one read count were filtered out as in panel *F*.

To better understand the coordination between Pol δ and Pol θ during TMEJ, Pol δ and Pol θ were purified (*SI Appendix,* Fig. S1*A*) and used to reconstitute TMEJ using DNA oligonucleotides representing 3′ single-stranded tails at double strand break sites. Pol θ with a shortened disordered linker (Pol θ Δcen) was used in most experiments due to the challenges in obtaining enough pure full-length enzyme. Both Pol θ Δcen and Pol δ are active DNA polymerases and able to fully extend a 16-mer primer annealed to a 30-mer template (Fig. 1 *A*, *Left*). When presented with single-stranded DNA, Pol δ displayed 3′ to 5′ exonuclease activity and Pol θ Δcen carried out stem loop extension via unimolecular self-pairing (Fig. 1 *A*, *Right*) (24). To directly observe TMEJ products, we designed two 90-mer oligonucleotides YZL1 and YZL2 labeled at the 5′ end with Cy5 or Cy3, respectively, which can pair at their 3′-terminal 6 bases to form a MH (Fig. 1*B*). After incubation of the oligonucleotides with Pol θ Δcen in reaction buffer with dNTPs, TMEJ products are readily identified on a native polyacrylamide gel by the merged white color (Fig. 1*B*, lanes 10 to 12). This protocol distinguishes TMEJ products from self-pairing products, which was not possible in studies using only ^32^P labeled DNA (25). YZL1 and YZL2 could also be extended separately by Pol θ Δcen via stem-loop self-pairing (Fig. 1*B*, lanes 2 to 4 and 6 to 8). We additionally tested shorter substrates such as 30-mer and 60-mer DNAs containing 6 bp terminal MH. All of them could be utilized by Pol θ Δcen for end-joining (*SI Appendix,* Fig. S2*A*).

Terminal MH rarely occur at DSBs in vivo; most MH used by Pol θ are usually embedded within 15 nucleotides from the 3′ end of ssDNAs at the break site (14, 15). To mimic this common situation, we designed another DNA substrate pair (YZL3 and YZL4) that can form a 6 bp MH with 4 additional nt at the 3′ end of YZL3 (Fig. 1*C* and *SI Appendix,* Fig. S3*A*). The 3′ end of YZL4 was modified with a phosphate group to block extension so that any extension originates only from YZL3. After incubation with Pol θ Δcen, TMEJ products were observed (Fig. 1*D*, lane 2 and *SI Appendix,* Fig. S3*A*, lanes 3 and 5). Other Cy5-labeled oligonucleotides with 4 bp MH and 0 to 2 additional unpaired bases yielded TMEJ products when paired to YZL4 (*SI Appendix,* Fig. S2*B*).

An equimolar mixture of Pol θ Δcen and Pol δ also generated TMEJ products in a reaction buffer containing dNTPs. These were formed with or without preincubation of polymerases with DNA before starting the reactions (Fig. 1*D*, lane 3 and *SI Appendix,* Fig. S3*A*, lane 4 and 6). The TMEJ products migrated similarly on gels following reactions with Pol θ Δcen alone, or in combination with Pol δ (Fig. 1*D* and *SI Appendix,* Fig. S3*A*). DNA sequencing was therefore necessary to determine MH choice and detect 3′ trimming (*SI Appendix,* Fig. S4*A*). Sequencing of TMEJ products showed that the designed 6 bp MH (GCAGGG) was not used by Pol θ Δcen when 4 additional unpaired nt (AGAT) were present on the top strand (Fig. 1*G* and *SI Appendix,* Fig. S4*B*). The TMEJ products were a mixture of different deletion sizes, showing that Pol θ Δcen can begin synthesis at many different MH anchoring positions on the template (Fig. 1*G* and *SI Appendix,* Fig. S4*B*). Sporadic point mutations, deletions, and insertions were observed in the products (*SI Appendix,* Fig. S4*B*). Many of these occurred in runs of identical pyrimidines or purines, a hallmark of low fidelity Pol θ-mediated DNA synthesis (26); other sequence changes might be due to PCR amplification or sequencing errors.

The most frequent MHs used by Pol θ had matched base pairing at the 3′ primer terminus, and often had mismatches within the 6 nt closest to the terminus, consistent with previously determined characteristics of MH selection by Pol θ (14). In reaction mixtures including Pol δ, most of the TMEJ products arose from events where YZL3 had been trimmed (Fig. 1*E* and *SI Appendix,* Fig. S3*B*), indicating that the exonuclease activity of Pol δ operates during the reaction. Trimming of 1-4 terminal nucleotides was most frequent (Fig. 1*F* and *SI Appendix,* Fig. S3*C*). After trimming of YZL3, Pol θ selects a MH that is different from that used for intact YZL3 (Fig. 1*G*). Notably, the designed 6 bp MH is not used by Pol θ Δcen unless the 4 terminal nt are trimmed by Pol δ, but trimming of 4 nt is not the predominant trimming outcome. Instead, Pol δ sometimes trims 0, 1, 2, or 3 nt, each giving rise to different anchoring positions (Fig. 1 *F* and *G* and *SI Appendix,* Fig. S3). These results show that Pol θ and Pol δ can work together in a simple reaction mixture to trim a 3′ end, locate a new MH, and extend from the 3′ end. After trimming, the selected internal MHs usually had matched base pairing at the 3′ primer terminus, and often still contained mismatches.

### Pol δ Exonuclease Affects MH Choice for the Initiation of TMEJ.

**Fig. 2.**
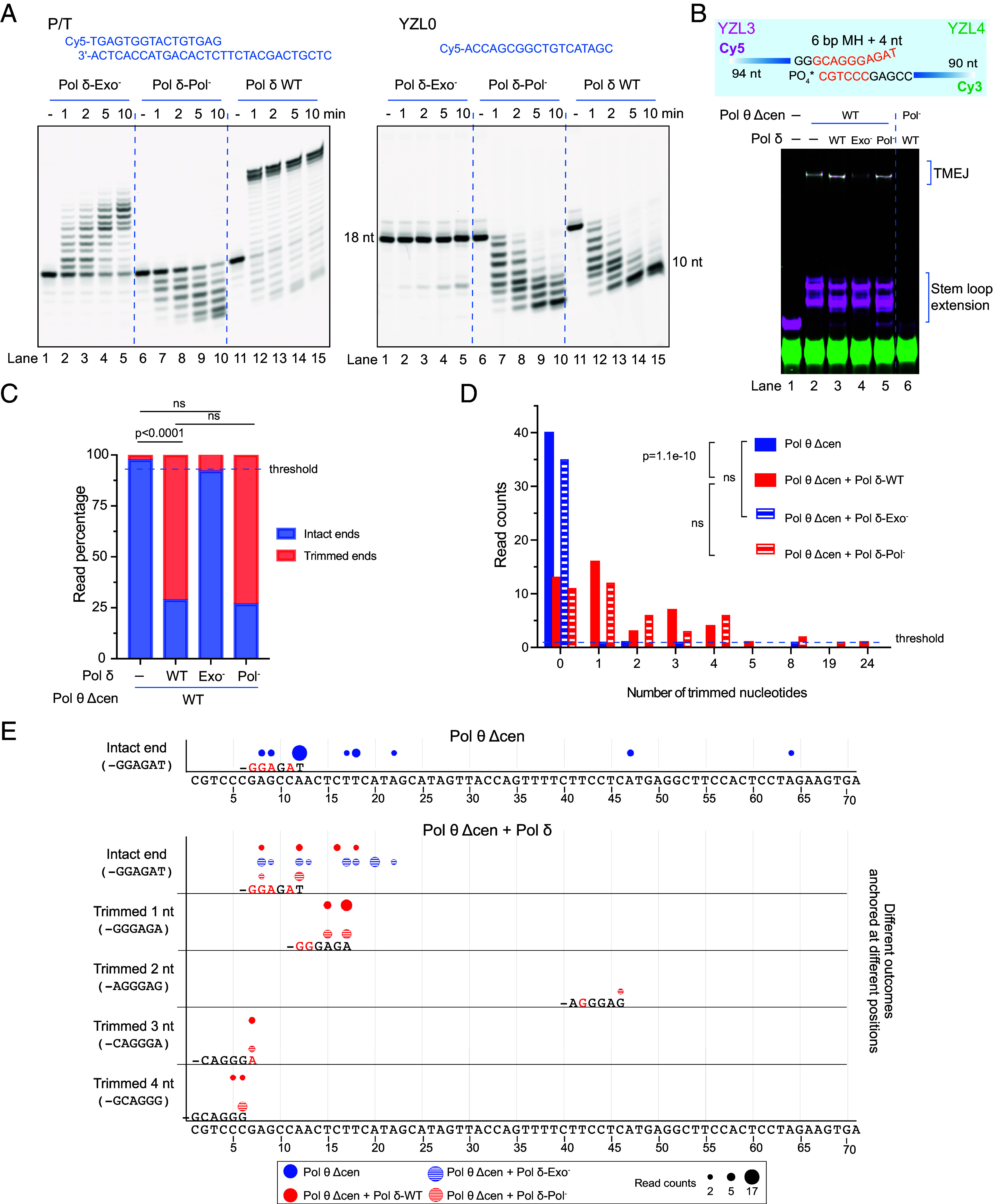
Pol δ exonuclease affects MH selection at the initiation of Pol θ mediated end-joining. (*A*) Activity of Pol δ and its variants on primer-template substrate (P/T) and ssDNA substrate YZL0. 25 nM DNA substrate was incubated with 25 nM Pol δ or its variants in the reaction mixture at 37 °C for indicated times. Reaction mixtures were separated by electrophoresis on a denaturing 15% polyacrylamide gel. (*B*) TMEJ with Pol θ Δcen or its variant Pol θ Δcen Pol^–^, and Pol δ or its variants Pol δ Pol^–^ or Pol δ Exo^–^. The schematic shows paired YZL3 and YZL4. The image shows reaction mixtures separated by electrophoresis on a native 10% polyacrylamide gel. 12.5 nM YZL3 and 12.5 nM YZL4 were incubated with 50 nM Pol θ Δcen, 50 nM Pol δ, and 100 µM dNTPs in reaction buffer at 37 °C for 15 min. (*C*) Read percentage of TMEJ outcomes arising from intact or trimmed ends of YZL3 in reaction products from (*B*). Outcomes arising from intact or trimmed ends are compiled from the distributions shown in panel *D*. The statistical significance is labeled with the *P* value derived from two-sided Fisher’s exact test. ns indicates no significant difference. (*D*) Read counts showing intact or trimmed nucleotide outcomes with YZL3 in reaction products from (*B*). The distributions are statistically different as indicated by the labeled *P* value derived from the Kolmogorov–Smirnov test (KS-test). The comparisons labeled “ns” have *P* > 0.5. (*E*) MH anchoring positions for the data in panels *C* and *D*. A circle marks each anchoring position of the YZL3 3′ end on the YZL4 template sequence, indicating the junction where two ssDNAs are joined by Pol θ. The YZL4 template sequence is shown at the bottom, numbered from the 3′ end. The size of the circle indicates the read count for each outcome. The different circles indicate reactions with Pol θ Δcen only, or with both Pol θ Δcen and Pol δ variants. Outcomes are categorized based on whether they resulted from an intact 3′ end or from a YZL3 3′ end trimmed by one to four nt. The most frequent MH is labeled under the corresponding circle. In the MH, bases in black are matched and bases in red are mismatched. Outcomes with only one read count were filtered out.

To better understand the roles of Pol δ polymerase and exonuclease activities in the initiation of TMEJ, we designed and purified protein variants (Fig. 4*A* and *SI Appendix,* Fig. S1 *B* and *C*). The Pol δ Exo^–^ variant replaces catalytic residue Asp 402 with Ala and is deficient in 3′ to 5′ exonuclease activity but has normal DNA polymerase activity (11, 27) (Fig. 2*A*). The Pol δ Pol^–^ variant replaces catalytic Asp 755 and Asp 757 with Ala and shows no DNA polymerase activity (28) but has normal exonuclease activity (Fig. 2*A*). We also purified a DNA polymerase-defective variant of Pol θ Δcen (Pol θ Δcen Pol^–^), replacing conserved catalytic Asp 2330 and Asp 2540 with Ala (*SI Appendix,* Fig. S1 *C* and *H* and Fig. 4*A*). Combining Pol θ Δcen with either Pol δ wild type or Pol δ Pol^–^ variant yielded TMEJ products with similar migration pattern on gels (Fig. 2*B*, lanes 3 and 5). The TMEJ and stem-loop products migrated slightly faster than products of reactions with Pol θ only (Fig. 2*B*, lanes 3 and 5 vs. lane 2). In the reaction with Pol θ Δcen and Pol δ Exo^–^ variant (Fig. 2*B*, lane 4), overall TMEJ product yield was decreased, suggesting that this variant interferes with TMEJ, as it does in vivo (11). As expected, no TMEJ products were formed by the Pol^–^ variant of Pol θ Δcen combined with Pol δ (Fig. 2*B*, lane 6). In fact, Pol δ digested almost all of the Cy5-labeled YZL3, while the 3′ phosphate on YZL4 conferred resistance to Pol δ exonuclease (Fig. 2*B*, lane 6).

DNA sequencing was used to determine the end-trimming pattern and MH selection for products of reactions with Pol θ and Pol δ variants. In reactions with Pol δ wild type and Pol^–^ variants, about 60% of products were trimmed (Fig. 2*C*), whereas no significant end-trimming occurred with Pol δ Exo^–^ variant (Fig. 2*C*). Reaction mixtures with Pol θ and exonuclease-competent Pol δ yielded products with either 0, 1, 2, 3, or 4 nt trimmed (Fig. 2*D**).* When the 3′ end remained intact (0 nt trimmed), the major anchoring positions were the same as found with Pol θ alone (Fig. 1*E*). More often, 1 to 4 nt were trimmed and distributions of selected MH were different in each case (Fig. 2*E*).

As observed with wild type Pol δ (Fig. 1*F*), Pol δ Pol^–^ usually trimmed one to four nucleotides from the 3′ end of the oligonucleotide that was used by Pol θ for end-joining (Fig. 2*E*). Anchoring positions were the same as in reactions containing Pol δ wild type or Pol δ Pol^–^ variant (Fig. 2*E*). These results show that editing of single-stranded DNA ends by Pol δ exonuclease influences the MH choice for Pol θ, and that Pol δ polymerase activity is not necessary during this step.

### ATPase and Strand-Capture Activities of the Pol θ HLD Facilitate TMEJ.

**Fig. 3.**
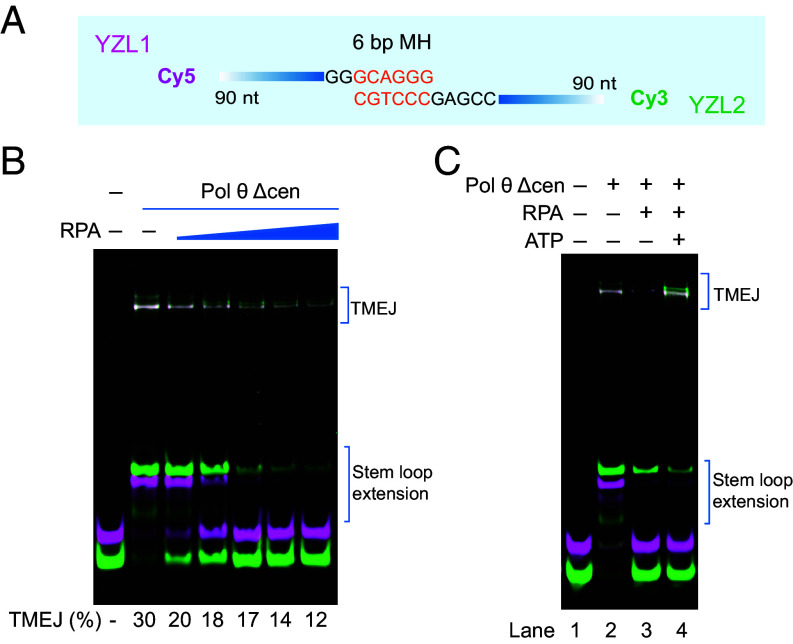
ATP addition rescues suppression of TMEJ by RPA. (*A*) Schematic of DNA substrate (YZL1 and YZL2) used in reactions with Pol θ Δcen, RPA, and ATP. (*B*) RPA suppression of TMEJ. 50 nM YZL1 and 50 nM YZL2 were preincubated with different concentrations of RPA at 37 °C for 10 min and then incubated with 100 nM Pol θ Δcen in reaction buffer at 37 °C for another 10 min. RPA concentrations were 0, 100, 300, 600, 900, and 1,200 nM. Reaction mixtures were separated by electrophoresis on a native 10% polyacrylamide gel. TMEJ percentage is labeled under each corresponding lane. (*C*) ATP rescues RPA suppression of TMEJ. 50 nM YZL1 and 50 nM YZL2 were preincubated with 600 nM RPA and 10 mM ATP at 37 °C for 10 min and then incubated with 200 nM Pol θ Δcen in reaction buffer at 37 °C for 15 min. Reaction mixtures were separated by electrophoresis on a native 10% polyacrylamide gel.

In cell nuclei, exposed single-stranded DNA is quickly coated and protected by RPA. RPA is known to suppress end-joining by purified Pol θ POL domain, and this suppression can be alleviated by an ATP-dependent function of separately purified Pol θ HLD (30). We found that end-joining mediated by Pol θ Δcen is also suppressed by purified RPA (*SI Appendix,* Fig. S1*D*) in the absence of ATP (Fig. 3 *A*–*C*). The addition of ATP alleviates this inhibition (Fig. 3*C* and *SI Appendix,* Fig. S5 *A* and *B*). MH anchoring beyond ~30 nt from the 3′ end is suppressed by RPA, shifting the distribution of MH pairing (*SI Appendix,* Fig. S6 *A–F*).

**Fig. 4.**
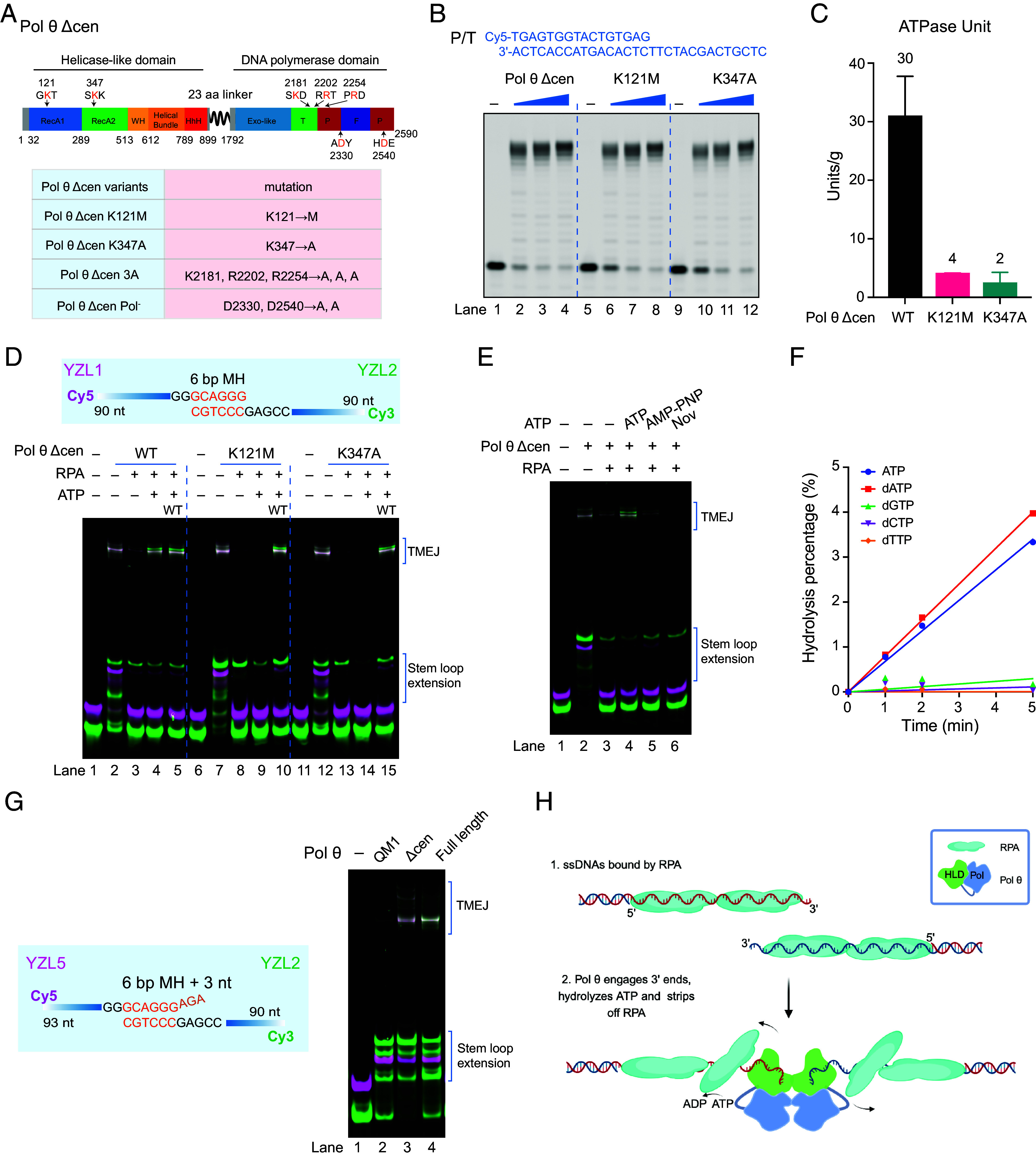
ATPase and strand capture activities of Pol θ HLD facilitate TMEJ. (*A*) Diagram of domains and constructs of human Pol θ Δcen. The HLD (POLQ 1-899) is connected to the polymerase domain (POL, POLQ 1792-2590) via a 23 amino acid linker. The locations of Pol θ Δcen variants are indicated. Subdomains are labeled with different colors [HLD: RecA1, RecA2, winged-helix (WH), Helical Bundle, helix–hairpin–helix (HhH), POL: Exonuclease-like, Thumb (T), Palm (P), Fingers (F)]. (*B*) Activity of Pol θ Δcen and its variants K121M and K347A with primer-template substrate (P/T). 20 nM DNA substrate was incubated with 0, 10, 20, or 40 nM Pol θ Δcen at 37 °C for 30 min. Reaction mixtures were separated by electrophoresis on a denaturing 15% polyacrylamide gel. (*C*) Specific activity of Pol θ Δcen and variants K121M and K347A. A unit is defined as the amount of Pol θ Δcen that catalyzes the production of 1 µmol of free phosphate per minute at pH 7.0 at 37 °C. The Perkin Elmer Easylite Kit was used with 3 nM M13mp18GTGx ssDNA (29), 5 µM ATP, and 40 nM Pol θ Δcen. (*D*) Pol θ Δcen variants K121M and K347A could not relieve RPA suppression of TMEJ. 50 nM YZL1 and 50 nM YZL2 were preincubated with 600 nM RPA, 10 mM ATP, and 15 mM MgCl_2_ at 37 °C for 10 min, then incubated with 200 mU Pol θ Δcen or variants K121M or K347A in 20 µL reaction buffer at 37 °C for 10 min, and with or without addition of 200 mU Pol θ Δcen WT for another 10 min at 37 °C. Reaction mixtures were separated by electrophoresis on a native 10% polyacrylamide gel. (*E*) RPA suppression of TMEJ is relieved by ATP but not by AMP-PNP or novobiocin. 50 nM YZL1 and 50 nM YZL2 were preincubated with 600 nM RPA, 10 mM ATP or AMP-PNP or 1 µM novobiocin and 15 mM MgCl_2_ at 37 °C for 10 min, then incubated with 200 nM Pol θ Δcen in reaction buffer at 37 °C for 10 min. Reaction mixtures were separated by electrophoresis on a native 10% polyacrylamide gel. (*F*) Pol θ Δcen hydrolyzes dATP as well as ATP. 3 nM M13mp18GTGx ssDNA, 1 mM ATP or dNTPs, and 40 nM Pol θ Δcen were incubated in reaction buffer (25 mM Tris pH = 7.4, 0.1 mg/mL BSA, 0.5 mM DTT, and 2.5 mM MgCl_2_) and measured with an ATPase/GTPase Activity Assay Kit. (*G*) TMEJ reaction with QM1, Pol θ Δcen or full-length Pol θ and ssDNA substrate. 25 nM YZL5 and 25 nM YZL2 were incubated with 30 mU QM1, Pol θ Δcen or full-length Pol θ in 15 µL reaction buffer at 37 °C for 20 min. Reaction mixtures were separated by electrophoresis on a native 10% polyacrylamide gel. (*H*) Working model for Pol θ HLD at the initiation of TMEJ. At the DNA double stand break sites, ssDNAs generated by nucleases are initially protected by RPA. The dimeric HLD of Pol θ uses its ATPase activity to displace RPA and captures two ssDNA tails. Figure created with BioRender.

The ATPase activity of the HLD is dependent on single-stranded DNA (22, 23). Pol θ HLD can use this activity to displace RPA in the 3′ to 5′ direction from single-stranded DNA (19). To determine whether the ATPase function is responsible for alleviating inhibition of TMEJ by RPA, we purified and tested two protein variants of Pol θ Δcen (*SI Appendix,* Fig. S1*E*). One of these changes Lys 121 in the conserved GKT motif to Met (Fig. 4*A*). An equivalent variant in HELQ greatly reduces ATPase activity and abolishes helicase activity (31). In the Pol θ HLD, the conserved Lys 121 residue is critical in the ATPase active site, making the protein contact with the nucleotide triphosphate tail (22). The K121M variant of Pol θ Δcen is active as a DNA polymerase (Fig. 4*B*) but is ATPase defective (7, 22, 32) (<15% activity, Fig. 4*C*). In reactions with Pol θ Δcen alone, this variant yields a TMEJ pattern similar to WT with various sets of DNA substrates (*SI Appendix,* Fig. S7 *A* and *B*). This single amino acid change, however, eliminates the ability of the protein to use ATP to reverse the inhibitory effects of RPA on TMEJ (Fig. 4*D*, lanes 3 and 4 vs. 8 and 9). Lys 347 (Fig. 4*A*) is predicted to be a DNA-binding residue by conservation with archaeal Hel308 (33, 34). Mutation of the equivalent Arg 255 residue in *S. solfataricus* Hel308 causes loss of activity (33). A cryo-EM structure of Pol θ HLD confirms that Lys 347 contacts a phosphate in the backbone of single-stranded DNA (9). We found that the K347A variant, although proficient in DNA polymerase activity (Fig. 4*B*) and yielding a TMEJ pattern similar to WT (*SI Appendix,* Fig. S7 *A* and *B*), also cannot reverse effects of RPA on TMEJ (Fig. 4*D*, lanes 13 and 14). The K347A variant is just as ATPase-deficient as the K121M variant (Fig. 4*C*), indicating that disabling DNA binding interferes with the conformational changes necessary to form the active site for efficient hydrolysis of ATP. Hydrolysis of nucleotide is necessary for RPA displacement as the nonhydrolyzable ATP analog AMP-PNP, unlike ATP, fails to rescue RPA-suppressed TMEJ (Fig. 4*E*). In the presence of dNTPs, TMEJ is not completely suppressed by RPA (Fig. 4 *D* and *E*) because Pol θ can hydrolyze dATP as well as ATP (Fig. 4*F*). Novobiocin, an inhibitor of Pol θ that blocks the DNA binding channel of the HLD (35, 36), completely suppresses the residual TMEJ in the presence of RPA and dNTPs (Fig. 4*E*, lanes 3 vs. 6). We conclude that DNA binding-dependent ATPase of the HLD is responsible for alleviating the RPA suppression in these reconstituted reaction mixtures (Fig. 4*H*).

RPA-coated ssDNA would also not be able to enter the Pol δ exonuclease channel, consistent with a modest inhibition of the Pol δ exonuclease by RPA (*SI Appendix,* Fig. S7 *C* and *E*). A high molarity of ATP also inhibits Pol δ exonuclease (*SI Appendix,* Fig. S7 *D* and *F*), probably by increasing the occupancy of the single-stranded DNA in the DNA polymerase site. Our experiments used 1 mM ATP for TMEJ reconstitution with Pol δ, where the Pol δ exonuclease is still highly active.

The HLD of Pol θ has a second function in TMEJ, distinct from the ATPase motor that displaces RPA. This function is “strand capture,” mediated by an HLD dimer that brings two single DNA strands into proximity (9, 18, 19), which should facilitate the joining of the 3′ ends. To assay for this function, the efficiency of TMEJ was compared using equal DNA polymerase units of Pol θ polymerase domain only (QM1) (16), Pol θ Δcen, and full-length Pol θ. With several different paired TMEJ substrates, the QM1 POL domain alone was much less efficient in producing TMEJ products compared to Pol θ Δcen or full-length Pol θ but the overall TMEJ pattern remained unchanged (Fig. 4*G* and *SI Appendix,* Fig. S8 *A*–*F*). This indicates that the strand capture activity of the Pol θ HLD facilitates the initiation of TMEJ (Fig. 4*H*).

### Pol θ Specific Primer Grasp Residues Are Important for Internal MH Anchoring.

**Fig. 5.**
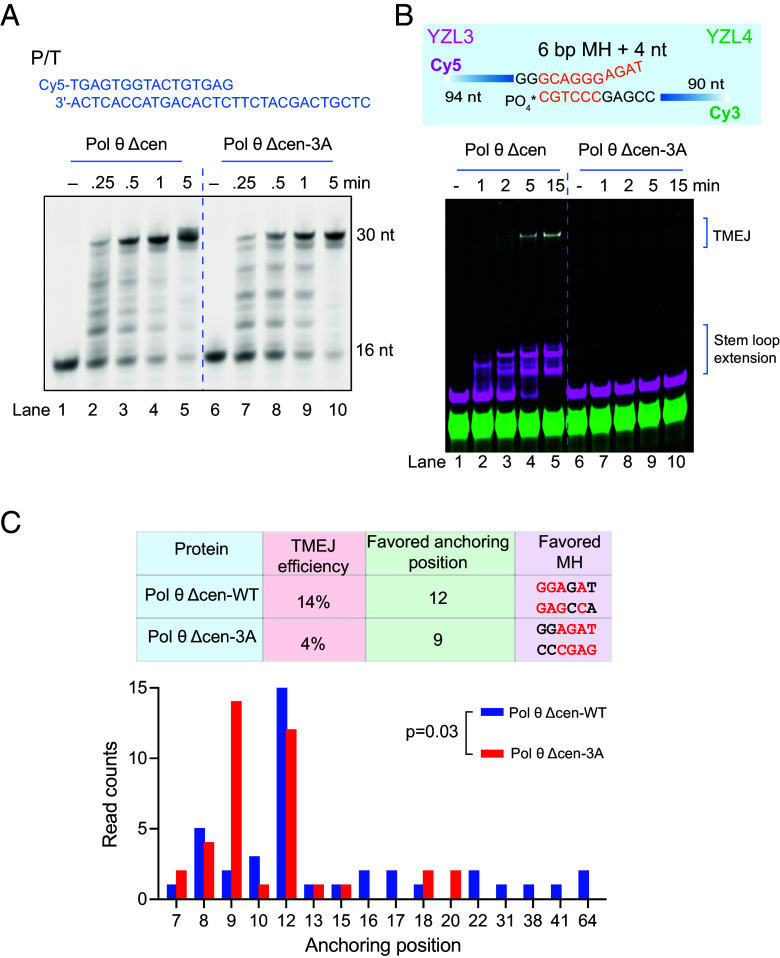
Primer-grasp amino acids of Pol θ POL are important for TMEJ. (*A*) Activity of Pol θ Δcen and its variant Pol θ Δcen-3A with primer-template substrate. 25 nM primer-template substrate (P/T) were incubated with 80 mU Pol θ Δcen or its variant 3A in 20 µL reaction buffer at 37 °C for the indicated time points. Reaction mixtures were separated by electrophoresis on a denaturing 15% polyacrylamide gel. (*B*) TMEJ reaction with Pol θ Δcen or variant 3A at different time points. 25 nM YZL3 and 25 nM YZL4 were incubated with 80 mU Pol θ Δcen or its variant 3A in 20 µL reaction buffer at 37 °C for the indicated time points. Reaction mixtures were separated by electrophoresis on a native 10% polyacrylamide gel. (*C*) Comparison of MH anchoring positions used by Pol θ Δcen and its variant 3A, obtained by sequencing and analysis of the TMEJ products in *B*, lanes 5 and 10.

Pol θ has an extraordinary ability to extend from short microhomologies during TMEJ, including MH interrupted by mismatches (14). The POL domain of Pol θ has five “primer-grasp” amino acids that contact the phosphate backbone of the primer (16, 37). Three are unique to Pol θ and not conserved in other A-family DNA polymerases. In human Pol θ, these amino acid residues (Lys 2181, Arg 2202, and Arg 2254) are needed for efficient bypass of damaged bases in template DNA (16) and unimolecular stem loop synthesis (24). We tested the influence of the primer-grasp residues on TMEJ with purified Pol θ. The Pol θ Δcen 3A variant (with three alanine substitutions at these positions, Fig. 4*A* and *SI Appendix,* Fig. S1*F*) showed polymerase activity comparable to wild type on a short primer-template substrate (Fig. 5*A*). The Pol θ Δcen 3A variant was unable to perform TMEJ efficiently on substrates with unpaired terminal nucleotide and showed reduced stem loop extension ability on ssDNA (Fig. 5*B* and *SI Appendix,* Fig. S9*A*) (24). The small amount of TMEJ product mediated by Pol θ Δcen 3A was isolated and sequenced to compare with products mediated by Pol θ Δcen WT. Pol θ Δcen 3A variant has a favored anchoring MH closer to the 3′ end (Fig. 5*C*). With a pair of oligonucleotides having a 6 bp matched terminal MH, Pol θ Δcen 3A could carry out TMEJ efficiently and suppress stem-loop extension (*SI Appendix,* Fig. S9*B*). These findings show that the three primer-grasp amino acids are important for TMEJ, assisting extension from internal MH.

### Functional TMEJ Reconstitution with Pol θ, Pol δ, and RPA.

**Fig. 6.**
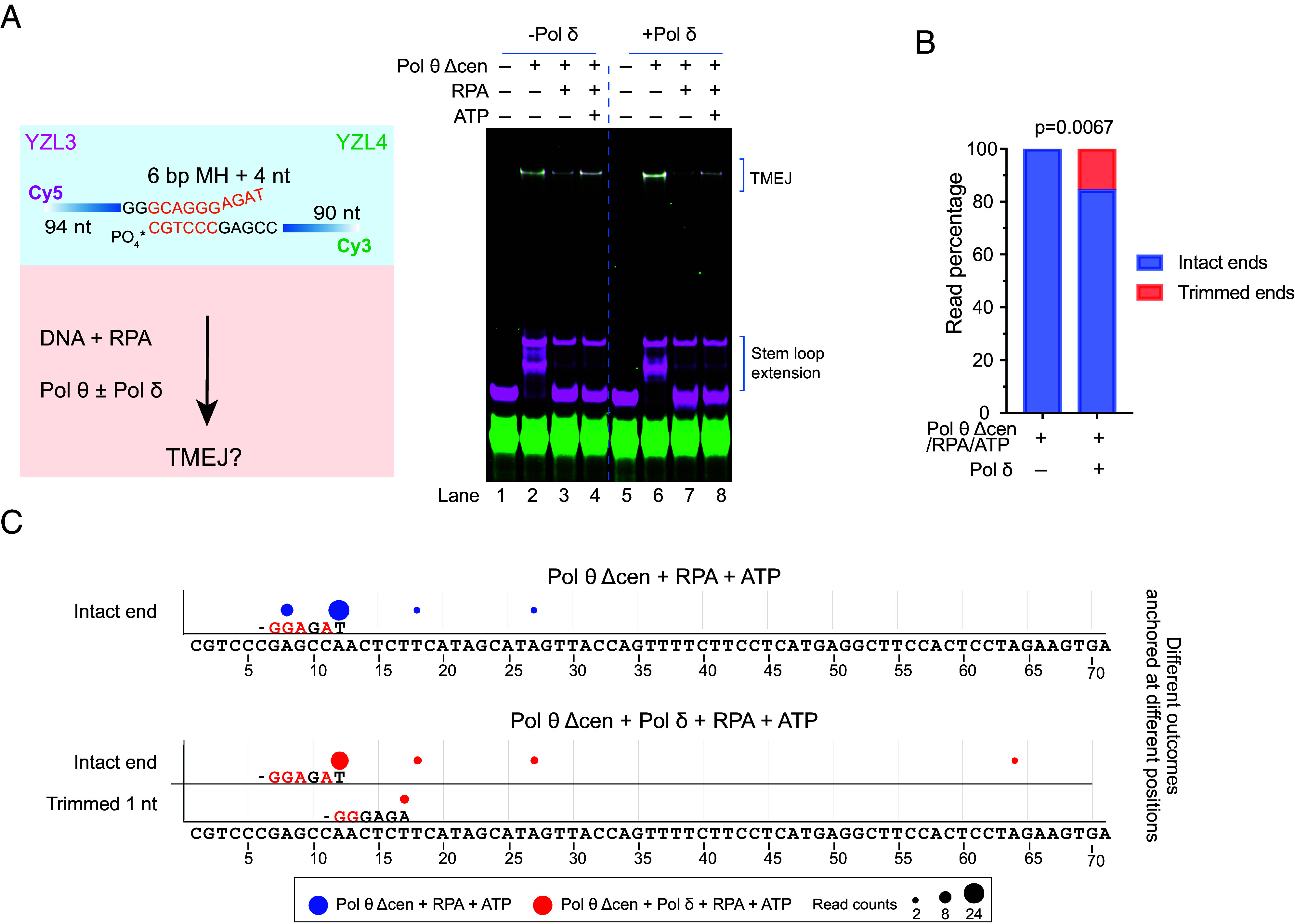
Functional TMEJ reconstitution. (*A*) TMEJ reconstitution with Pol θ Δcen, Pol δ, RPA, ATP, and DNA substrate. 25 nM YZL3 and 25 nM YZL4 were preincubated with 300 nM RPA and 6 mM MgCl_2_ at 37 °C for 10 min and then incubated with different combinations of 100 nM Pol θ Δcen, 100 nM Pol δ, 1 mM ATP, and 50 µM dNTPs in reaction buffer at 37 °C for 15 min. Reaction mixtures were separated by electrophoresis on a native 10% polyacrylamide gel. (*B*) Read percentage of TMEJ outcomes arising from intact or trimmed ends of YZL3 from samples in panel A (lanes 4 and 8). The statistical significance is labeled with the *P* value derived from two-sided Fisher’s exact test. (*C*) MH anchoring positions for the data in panel *B*. A circle marks each anchoring position of the 3′ end of YZL3 on the YZL4 template sequence. This is the junction where the two ssDNAs are joined by Pol θ. The YZL4 template sequence is shown at the bottom, numbered from the 3′ end. The size of the circle indicates the read count for each outcome. The blue circles are for reactions with Pol θ Δcen (with RPA and ATP), and the red circles for reactions with both Pol θ Δcen and Pol δ (with RPA and ATP). Outcomes are categorized based on whether they resulted from an intact 3′ end or from a YZL3 3′ end trimmed by one nt. The most frequent MH is labeled under the corresponding circle. In the MH, bases in black are matched and bases in red are mismatched. Outcomes with only one read count were filtered out.

To determine whether TMEJ can be carried out with all the purified components considered here, we preincubated ssDNA substrates with RPA and MgCl_2_. These were then mixed with Pol θ Δcen, Pol δ, dNTPs, and ATP to initiate the reaction. In these reactions, RPA suppressed end joining and ATP rescued this suppression (Fig. 6*A*, lanes 1 to 4). This was also true in the presence of Pol δ (Fig. 6*A*, lanes 5 to 8). Sequencing of the products shows that Pol δ was functional in the reaction, as some TMEJ events were generated from Pol δ-digested DNA substrates (Fig. 6*B*). The MH selected by Pol θ after Pol δ trimmed 1 nt (Fig. 6*C*), corresponds to the major MH selected after 1 nt trimming in TMEJ reactions with Pol θ with Pol δ in the absence of RPA and ATP (Fig. 1*G*). Because trimming frequency was low in the presence of RPA, we tested a different pair of oligonucleotides. Multiple trimming products were observed at a low frequency (*SI Appendix*, Fig. S10). The suppression of Pol δ exonuclease activity by RPA (*SI Appendix*, Fig. S7 *C* and *E*) may account for the lower fraction of trimming by Pol δ in the reaction with RPA (Fig. 6*B* and *SI Appendix*, Fig. S10). To test whether the length of the central domain of Pol θ might affect the switching between Pol θ and Pol δ, we tested two variants of Pol θ Δcen purified with expanded central domains (*SI Appendix*, Figs. S1*G* and S11*A*). All three variants produced a low frequency of TMEJ products in the presence of RPA, but a longer central domain did not increase the fraction of trimmed products (*SI Appendix*, Fig S11 *B–D*). In all cases, Pol θ, Pol δ, RPA, and ATP could function together in a reconstituted reaction.

## Discussion

### Pol δ Excises One Nucleotide at a Time Rather than Removing a Flap at the Initiation of Pol θ-Mediated End Joining.

By reconstituting TMEJ in vitro with purified proteins and DNA oligonucleotides, it has been possible to better define the sequential involvement and contributions of each component during the initiation steps of TMEJ.

In mammalian cells, Pol δ exonuclease is involved in removing unpaired nucleotides flanking a MH (11). It has been suggested that Pol δ might act during TMEJ by “flap cutting,” eliminating unpaired nucleotides en bloc beyond the MH (2, 11, 38). Flap cutting is not a plausible mechanism, however, because Pol δ exonuclease cannot gain access to DNA near the MH when it is bound by Pol θ. In editing sites of DNA polymerases, three or four nucleotides of single-stranded DNA are located within the exonuclease channel to allow trimming of the terminal nucleotide (39) (*SI Appendix,* Fig. S12*A*), and 5 to 6 more nucleotides are bound by other areas of the Pol δ enzyme (39, 40). Similarly, at least 10 bp of both template and primer strands are bound within Pol θ (16, 41, 42).

The best characterized function of the Pol δ exonuclease is the editing of DNA polymerase errors during DNA replication. When a base is misincorporated (at a frequency of ~ 10^−5^ by Pol δ) (43), the primer is transferred from the DNA polymerase site to the exonuclease site, where the terminal nucleotide is removed. The primer strand is then transferred back to the polymerase active site (44–46). The results described in the present study are consistent with this action and indicate that after trimming the terminal nucleotide, the edited strand may transfer back to Pol θ. Switching between the editing site of Pol δ and the polymerization site of another polymerase has been proposed for semiconservative DNA replication, to explain how Pol δ edits misincorporations introduced by Pol ϵ (47) and Pol α (48). Once the editing of a nucleotide occurs within the Pol δ exonuclease site, the single strand may return briefly to the Pol δ polymerase site, but Pol δ lacks the ability to extend from a very short MH. Only a transfer to Pol θ will be productive.

Multiple DNA polymerases with proofreading ability, including human Pol ϵ (44), human Pol γ (49, 50), T4 DNA polymerase (51), and *Escherichia coli* DNA polymerase III (52), excise one nucleotide at a time. After trimming a base, the trimmed primer must be completely withdrawn from its exonuclease site, allowing the dNMP product to diffuse out of the narrow exonuclease channel (49). This is likely facilitated by the net negative charge in the exonuclease channel as shown by the Pol γ structure (*SI Appendix,* Fig. S12*B*), which will reduce the affinity to single-stranded DNA following terminal nucleotide excision (50). The nonprocessive nature of the Pol δ exonuclease reaction is consistent with its slow reaction on single-stranded DNA oligonucleotides (Figs. 1*A* and 2*A* and *SI Appendix,* Fig. S7 *C* and *D*). The single-stranded oligonucleotide can be trimmed down to a minimum of about 10 nt; shorter oligonucleotides apparently cannot feed through the exonuclease channel because they lack sufficient contact with the protein (*SI Appendix,* Fig. S12 *A* and *B*).

As Pol δ edits one nucleotide at a time, trimming of more than one nucleotide requires multiple cycles of exit and entry into the exonuclease channel. Removing the 3′ nucleotide yields a new terminus and thereby unlocks a new set of MH anchoring positions for Pol θ.

There is evidence that the APE2 nuclease can also function during TMEJ in mammalian cells (38). The flap endonuclease activity of APE2 would not be relevant to TMEJ, for the reasons discussed above, but it is plausible that the 3′ to 5′ exonuclease activity of APE2 could substitute for the equivalent function of Pol δ. The Pol δ polymerase activity is not necessary for the initiation of end-joining in the present study, but it works later in the TMEJ process. After Pol θ synthesizes a short stretch of nucleotides (11), a primer-template structure is generated that is suitable for extension by Pol δ.

### Functional TMEJ Is Reconstituted with Pol δ, Pol θ, RPA, and DNA Substrate.

**Fig. 7.**
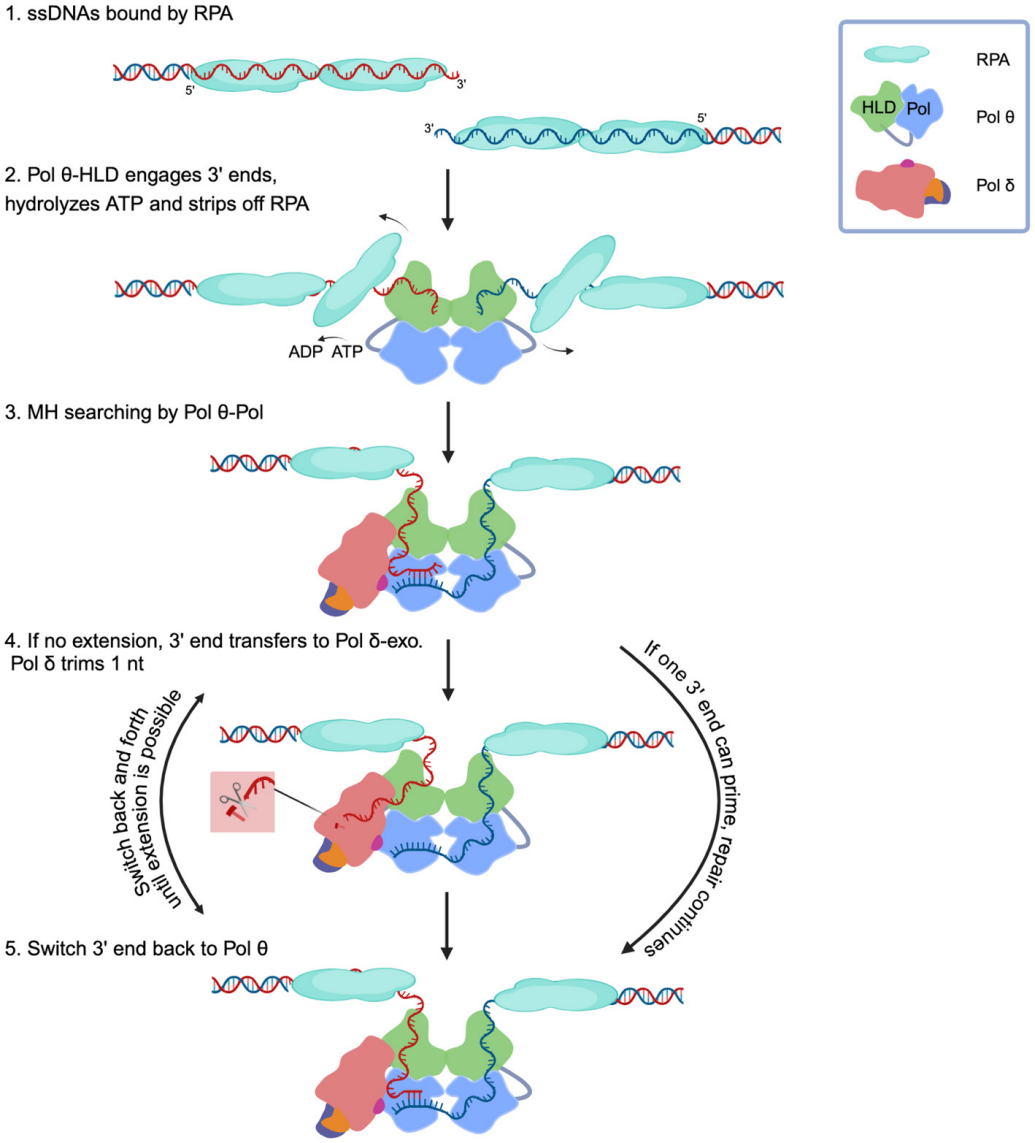
Transfer of DNA between Pol θ and Pol δ resets MH choice during TMEJ. At the site of a DNA double strand break, DNA resection nucleases generate ssDNA tails with terminal 3′ ends. (1) These tails are quickly protected by RPA (cyan in the figure, about 30 nt per RPA heterotrimer). (2) The HLD of Pol θ (green) uses its ATPase activity to displace RPA and capture two strands of DNA, each held by a subunit of the dimeric helicase. The 3′ regions of the strand are in proximity and may transiently pair at a short MH. (3) These DNA strands are available for binding by the POL domain (blue) of Pol θ. Pol θ can extend from an appropriately paired short MH. (4) If extension does not take place, the primer strand can be transferred to the Pol δ exonuclease site (salmon), where the terminal nucleotide is removed. (5) The strand is then transferred back to the POL domain of Pol θ, resetting the MH search. Extension takes place if a suitably placed MH is located. If a new anchoring position is not found, the DNA may again be transferred from Pol θ to Pol δ exonuclease to trim another nucleotide. Several cycles of this switching between Pol θ and Pol δ may take place. A physical interaction between the polymerases may facilitate the switch. Figure created with BioRender.

Reconstitution in vitro is a powerful tool to study the coordination of proteins in a repair pathway. The experiments reported here, suggest a model for the initial steps of TMEJ (Fig. 7). At the site of a DNA double strand break, nucleases resect the DNA and generate 3′ ssDNA tails. Such DNA is initially bound and protected by RPA in cells. It is established that the HLD is essential for TMEJ in vivo (30, 53, 54). Our experiments underline two distinct functions of Pol θ HLD. The dimeric HLD captures two ssDNA tails so that the 3′ ends are in proximity and may transiently pair at a short MH. During this process Pol θ uses its ATPase activity to displace RPA or other single-stranded DNA binding proteins.

The ssDNA is then available for binding by either the POL domain of Pol θ or Pol δ. Pol θ (but not Pol δ) can extend from a short MH when the 3′ end is paired (14). Pol θ cannot efficiently anchor some microhomologies because of excessive mismatches or distance from the 3′ end. If extension from the 3′ end is not possible, the ssDNA will enter the exonuclease site of Pol δ to remove the terminal nucleotide. After transfer back to Pol θ, the newly generated terminal sequence may then transiently pair at a MH, possibly at a different position than in the first iteration. The POL domain of Pol θ will extend the 3′ end, if it is suitably paired. If not, the strand transfers back to the Pol δ exonuclease where another nucleotide is trimmed. The back-and-forth transfer of DNA between these two polymerases may be facilitated by a previously described physical interaction (11), and by additional coordinating proteins. Several cycles of this switching between Pol θ and Pol δ may take place before a MH is selected.

## Materials and Methods

### Proteins and Oligonucleotides.

The cDNA sequence for human Pol θ Δcen was codon optimized for mammalian expression by GenScript Biotech, and subcloned into phCMV1-2XMBP plasmid. The protein was transfected into human Expi293F cells using polyethylenimine (PEI) (24765, Polysciences) solution. After 48 h, the overexpressed protein was purified as previously described (55). Pol θ variants Δcen K121M, Δcen K347A, Δcen Pol^–^ (D2330/2540A), Δcen 3A (K2181A/R2202A/2254A), Δcen190, and Δcen247 were derived and purified in a similar manner. Recombinant human Pol δ holoenzyme and its variants Pol δ Exo^–^ (D402A), Pol δ Pol^–^ (D755/757A) were purified as described (56). RPA was purified according to (57). Klenow Fragment (3′→5′ exo^–^) (M0212L) was purchased from New England Biolabs. All oligonucleotides (Sequences listed in *SI Appendix,* Table S1) were synthesized by Integrated DNA Technologies.

### Biochemical Reactions.

Functional TMEJ mixtures contained 25 nM each of two oligonucleotides, one 5′-labeled with Cy5 and one with Cy3. The oligonucleotides were first incubated with 300 nM RPA for 10 min at 37 °C in 5 µL reaction buffer A [25 mM potassium phosphate, pH 7, 0.1 mg/mL bovine serum albumin (BSA), 5 mM dithiothreitol (DTT), and 6 mM MgCl_2_]. This mixture was then added to a 5 µL mixture of 100 nM Pol θ Δcen and 100 nM Pol δ in reaction buffer B [25 mM potassium phosphate, pH 7, 0.1 mg/mL BSA, 5 mM DTT, 50 µM dNTPs, 1 mM ATP (A7699, Sigma)]. After 15 min incubation at 37 °C, 2 µL 6X stop buffer [300 mM Tris-HCl pH 7.5, 3 mg/mL Proteinase K (P8107S, New England Biolabs), 120 mM EDTA, and 1.2% SDS] was added and incubated for 10 min at 37 °C to digest the proteins. Reactions were mixed with 3 µL 6X native gel loading buffer (40% sucrose with 0.025% bromophenol blue) and 7 µL samples were loaded onto a native polyacrylamide gel (10% 19:1 acrylamide:bis-acrylamide, 0.5X TBE buffer, 0.075% APS, and 0.0375% TEMED) and run at 5 W/gel using Bio-Rad PROTEAN II XL Gel Running System. Results were obtained by Amersham Typhoon 5 (Cytiva) scanning fluorescence signal on gels. For other TMEJ reactions, the concentrations of enzyme and oligonucleotides were varied and labeled in the figure legends. For primer-template extension and ssDNA degradation testing, after reaction at 37 °C, an equal amount of 2X denaturing stop buffer (20 mM EDTA in 95% formamide containing bromophenol blue) was added and boiled at 95 °C for 5 min. 10 µL reactions were loaded onto a denaturing polyacrylamide gel (15% 19:1 acrylamide:bis-acrylamide, 7 M urea, 0.5X TBE buffer, 0.075% APS, and 0.0375% TEMED) and run at 5 W/gel using Bio-Rad PROTEAN II XL Gel Running System. ImageJ was used to measure the intensity of individual TMEJ bands and whole lane products. The percentage of TMEJ is the intensity of TMEJ bands divided by the intensity of the whole lane products × 100.

### Sequencing Experiments.

TMEJ sequencing was done as described (14). Briefly, TMEJ reaction samples were cleaned up with a MinElute PCR purification Kit (QIAGEN, 28004) and then amplified by NEBNext^®^ UltraTM II Q5^®^ Master Mix (M0544L, New England Biolabs) and primers R/F for 10 to 19 cycles. After gel cleanup with the QIAquick Gel Extraction Kit (QIAGEN, 28004), amplicons were assembled into pUC19 vector with NEBuilder® HiFi DNA Assembly Master Mix (E2621L, New England Biolabs). The assembled vectors were transformed into chemically competent *E. coli* TOP10 cells. After overnight culture at 37 °C, 50 colonies were sent to Genewiz (South Plainfield, NJ) or Molecular Cloning Laboratories (South San Francisco, CA) for Sanger sequencing with primer F-Seq. Sequencing results were analyzed by ClustalW alignment with reference sequence via MacVector software.

### Polymerase and ATPase Specific Activity Assay.

The specific polymerase activity of QM1, Pol θ Δcen, and its variants K121M, K347A, 3A, and full-length Pol θ were measured and calculated with EvaEZ^TM^ Fluorometric Polymerase Activity Assay Kit (29051, Biotium) as previously described (24). The specific ATPase activity of Pol θ Δcen and its variants K121M, K347A were measured and calculated with the Perkin Elmer Easylite Kit (6066741). The ATP hydrolysis assay of ATP and dNTPs was measured with ATPase/GTPase Activity Assay Kit (Sigma-Aldrich, MAK113).

## Supplementary Material

Appendix 01 (PDF)

Dataset S01 (XLSX)

## Data Availability

All study data are included in the article and/or supporting information.
