# Supplementary material for: Coordinated transfer of DNA between Pol θ and Pol δ resets microhomology choice during double-strand break repair
Source: Proc Natl Acad Sci U S A. 2025 Nov 19;122(47):e2513018122. doi: 10.1073/pnas.2513018122 (PMC12663940; doi:10.1073/pnas.2513018122)
Supplement: Supplementary file 1 — Appendix 01 (PDF) [file pnas.2513018122.sapp.pdf]

PNAS

Supporting Information for

Coordinated transfer of DNA between Pol θ and Pol δ resets microhomology choice during double-strand break repair

Yuzhen Li[a], Mark Returan[a], Adele T. Guerin[a], April M. Averill[b], Dorcas Oladapo[a], Sylvie Doublié[b] and Richard D. Wood[a,1]

[a]Department of Epigenetics and Molecular Carcinogenesis, The University of Texas MD Anderson Cancer Center, Houston, TX, 77230
[b]Department of Microbiology and Molecular Genetics, University of Vermont, Burlington, VT, 05405

[1]Corresponding author: Richard D. Wood
Email: rwood@mdanderson.org

This PDF file includes:

Figures S1 to S12
Table S1

Fig S1

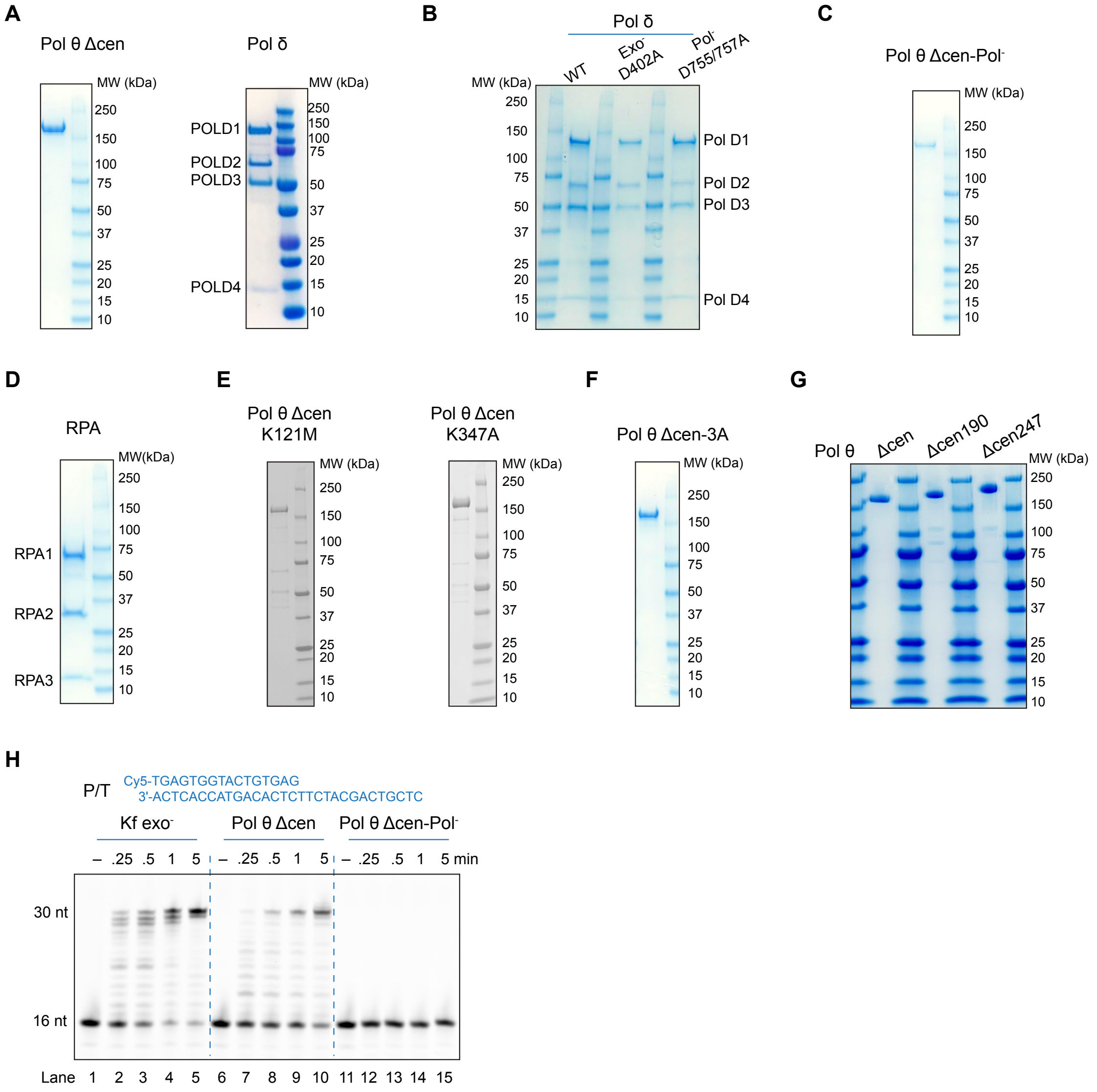

Figure S1. Protein purification and activity.

Polyacrylamide gels showing purified (A) human Pol θ Δcen and Pol δ holoenzyme (subunits Pol D1 - 4), (B) Pol δ variants Exo⁻ and Pol⁻, (C) Pol θ Δcen Pol⁻, (D) RPA (subunits RPA1 - 3), (E) Pol θ Δcen K121M and K347A, (F) Pol θ Δcen-3A, (G) Pol θ Δcen190 and Pol θ Δcen247. Pol θ Δcen190 adds Pol θ residues 1289-1467 to the Pol θ Δcen construct. Pol θ Δcen247 adds Pol θ residues 1289-1524 to the Pol θ Δcen construct.

(H) Activity of Pol θ Δcen and its variant Pol θ Δcen Pol⁻ on primer-template substrate. Exonuclease defective *E. coli* DNA polymerase I Klenow fragment (Kf exo⁻) was used as control. 25 nM primer-template substrate (P/T) was incubated with 4 mU/μL Kf exo⁻ or 25 nM Pol θ Δcen or its variant Pol θ Δcen Pol⁻ at 37 °C for the indicated times.

Fig S2

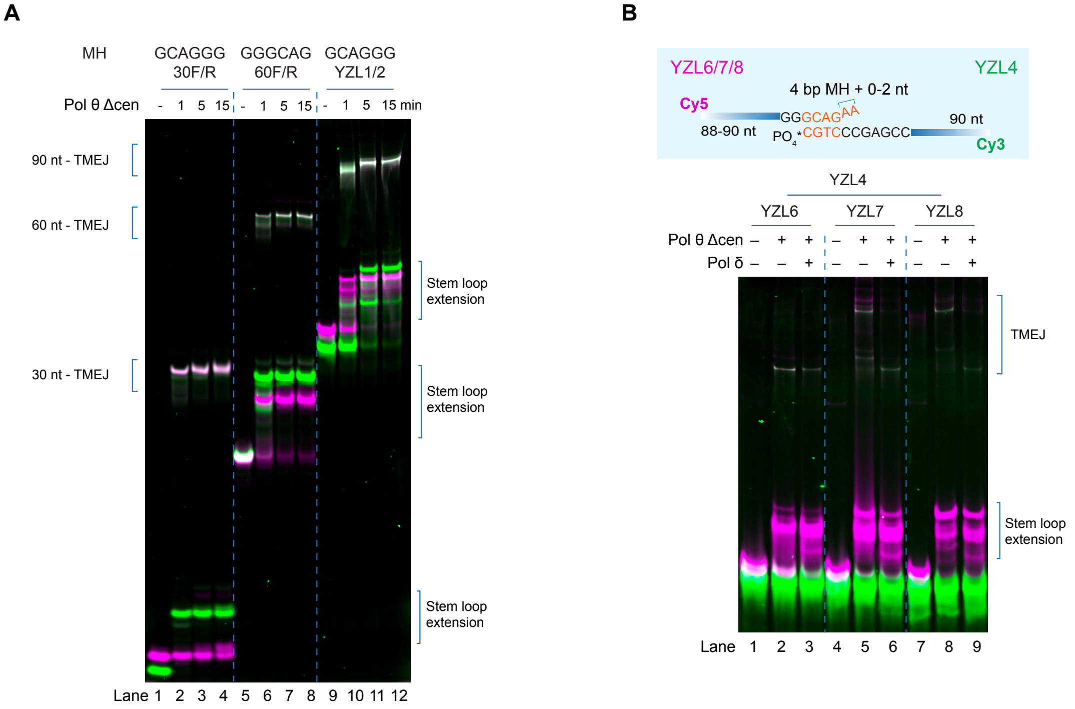

Figure S2. Pol θ and Pol δ work together on different TMEJ substrates.

(A) TMEJ with Pol θ Δcen and ssDNA substrates of different lengths. 50 nM of two different ssDNAs were incubated with 200 nM Pol θ Δcen at 37 ºC for the indicated times. 30-mer (30F/R), 60-mer (60F/R) and 90-mer (YZL1 and YZL2) substrates contain a terminal 6 bp MH. The terminal MH sequences are labeled at the top of the figure. Reaction mixtures were separated by electrophoresis on a native 10% polyacrylamide gel.

(B) TMEJ with Pol θ Δcen, Pol δ and ssDNA substrates which contain a designed 4 bp MH followed by 0 - 2 unpaired nucleotides. 12.5 nM YZL6/7/8 and 12.5 nM YZL4 were incubated with 50 nM Pol θ Δcen/50nM Pol δ at 37 ºC for 15 min. Reaction mixtures were separated by electrophoresis on a native 10% polyacrylamide gel.

Fig S3

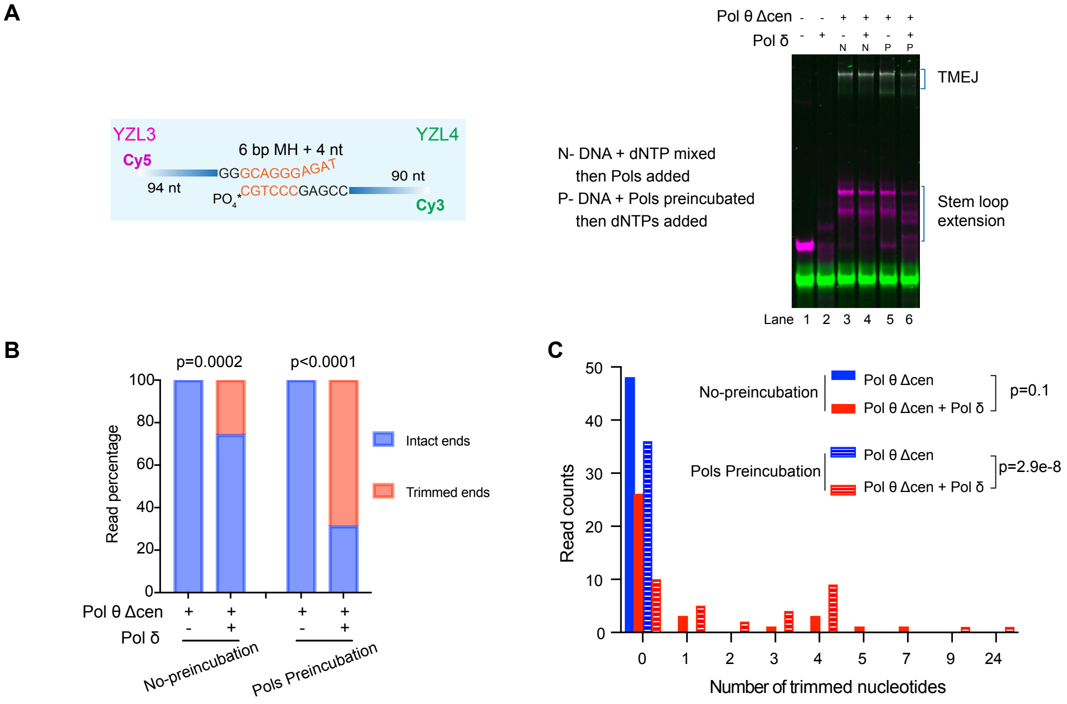

Figure S3. Pol θ can use Pol δ trimmed oligonucleotides as end-joining substrates.

(A) TMEJ with Pol θ Δcen, Pol δ and paired ssDNA substrate. The schematic shows paired DNA substrate (YZL3 and YZL4) containing 5' fluorescent labels and a 6 bp MH followed by 4 unpaired bases. The 3' end of YZL4 was modified with a phosphate group to block extension and digestion. Reaction mixtures were separated by electrophoresis on a native 10% polyacrylamide gel. N indicates the DNA substrates were mixed with dNTPs before starting the reaction by adding Pol θ Δcen and Pol δ, while P indicates the DNA substrates were pre-incubated with Pol θ Δcen and Pol δ first for 10 min before starting the reaction by addition of dNTPs. Reactions contained 12.5 nM YZL3, 12.5 nM YZL4, 25 nM Pol θ Δcen, 50 nM Pol δ and 100 uM dNTPs and were incubated at 37 ^{o}C for 30 min.

(B) Read percentage of TMEJ outcomes arising from intact or trimmed ends of YZL3 in reaction products from (A). The statistical significance is labeled with the p value derived from two-sided Fisher’s exact test.

(C) Read counts showing intact or trimmed nucleotide outcomes with YZL3 in reaction products from (A). The statistical significance is labeled with the p value derived from Kolmogorov-Smirnov test (KS-test).

Fig S4

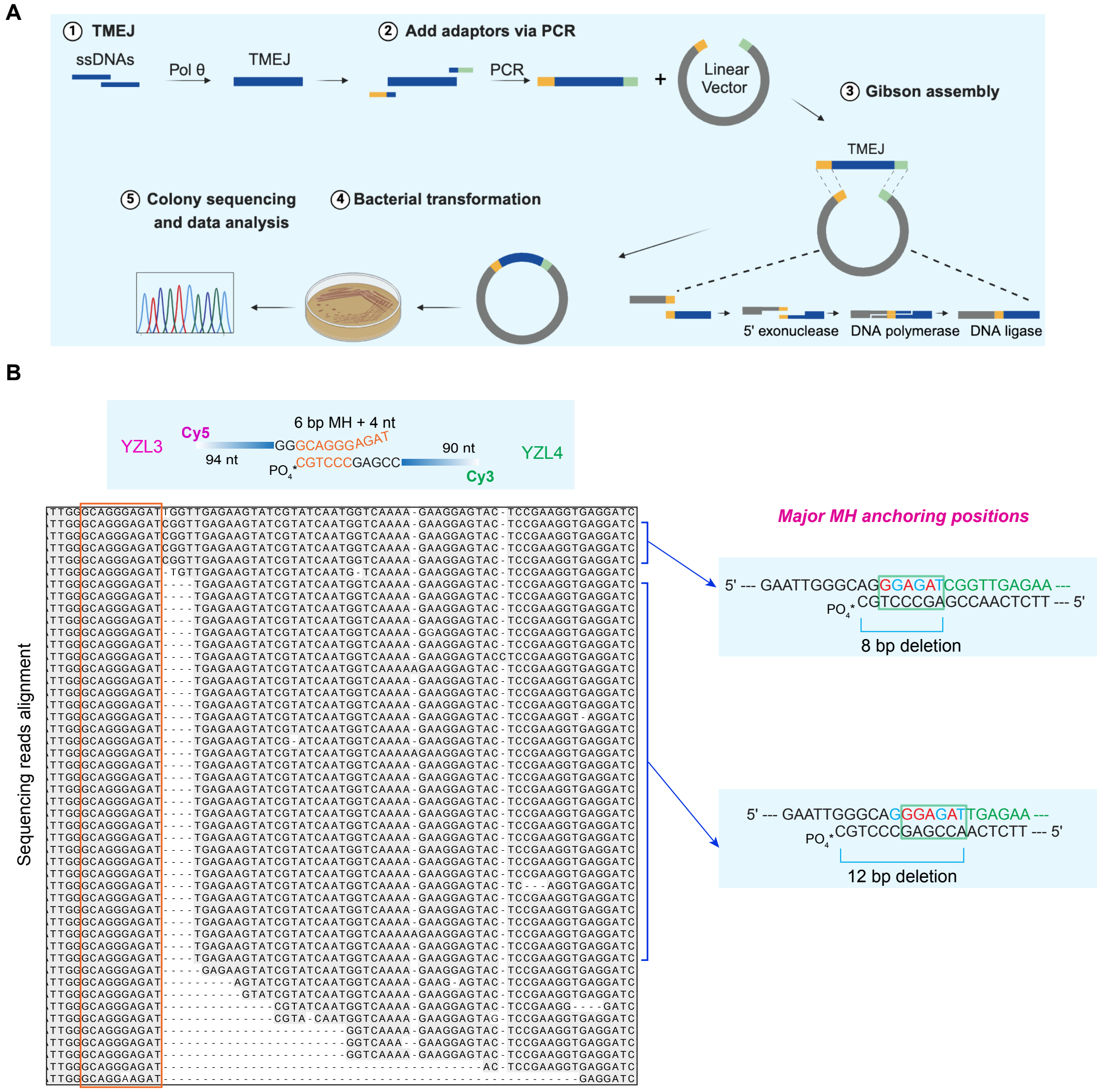

Figure S4. DNA sequencing to characterize TMEJ products.

(A) DNA sequencing sample preparation to characterize TMEJ products. PCR amplified TMEJ products were transferred to pUC19 vector via Gibson assembly. Assembled plasmids were transformed into *E. coli* and Sanger sequencing was performed for individual colonies. Figure created with BioRender.

(B) Sequence alignment and major MH anchoring positions of TMEJ products formed by Pol θ Δcen joining YZL3 and YZL4. DNA sequence reads for the top strand are shown. The orange box in the alignment labels the 3' end of YZL3. TMEJ products are initiated at different anchoring positions as illustrated by gaps in the alignment. The most frequent anchoring positions (deletion sizes) are shown at the right, with details of local pairing information in the MHs used by Pol θ Δcen. In the diagrams of major MH anchoring positions, the green square labels 6 bp of the local MH sequence environment. Red nucleotides indicate mismatches and blue ones indicate matches. Nucleotides are shown in green following the microhomology region, to indicate the start of the region newly synthesized by Pol θ Δcen.

Fig S5

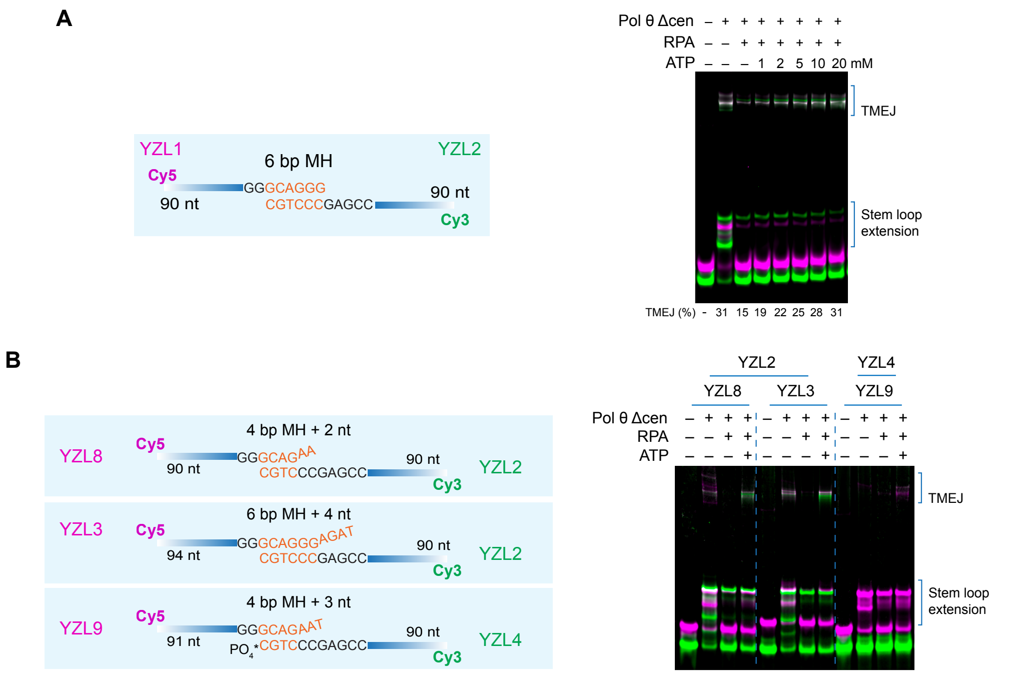

Figure S5. Effect of RPA and ATP on TMEJ.

(A) Dependence on ATP concentration for rescue of RPA suppression in TMEJ with Pol θ Δcen. 50 nM YZL1 and 50 nM YZL2 were pre-incubated with 600 nM RPA, the indicated concentration of ATP-$MgCl_2$ complex, and an additional 5 mM $MgCl_2$ at 37 °C for 10 min and then incubated with 100 nM Pol θ Δcen in reaction buffer at 37 °C for 10 min. Reaction mixtures were separated by electrophoresis on a native 10% polyacrylamide gel. TMEJ percentage is labeled under corresponding lanes.

(B) ATP rescues RPA suppression of TMEJ with Pol θ Δcen and substrates YZL8/YZL2, YZL3/YZL2 and YZL9/YZL4. 50 nM of each ssDNAs were pre-incubated with 600 nM RPA, 15 mM $MgCl_2$, and 10 mM ATP at 37 °C for 10 min and then incubated with 200 nM Pol θ Δcen in reaction buffer at 37 °C for 10 min. Reaction mixtures were separated by electrophoresis on a native 10% polyacrylamide gel.

Fig S6

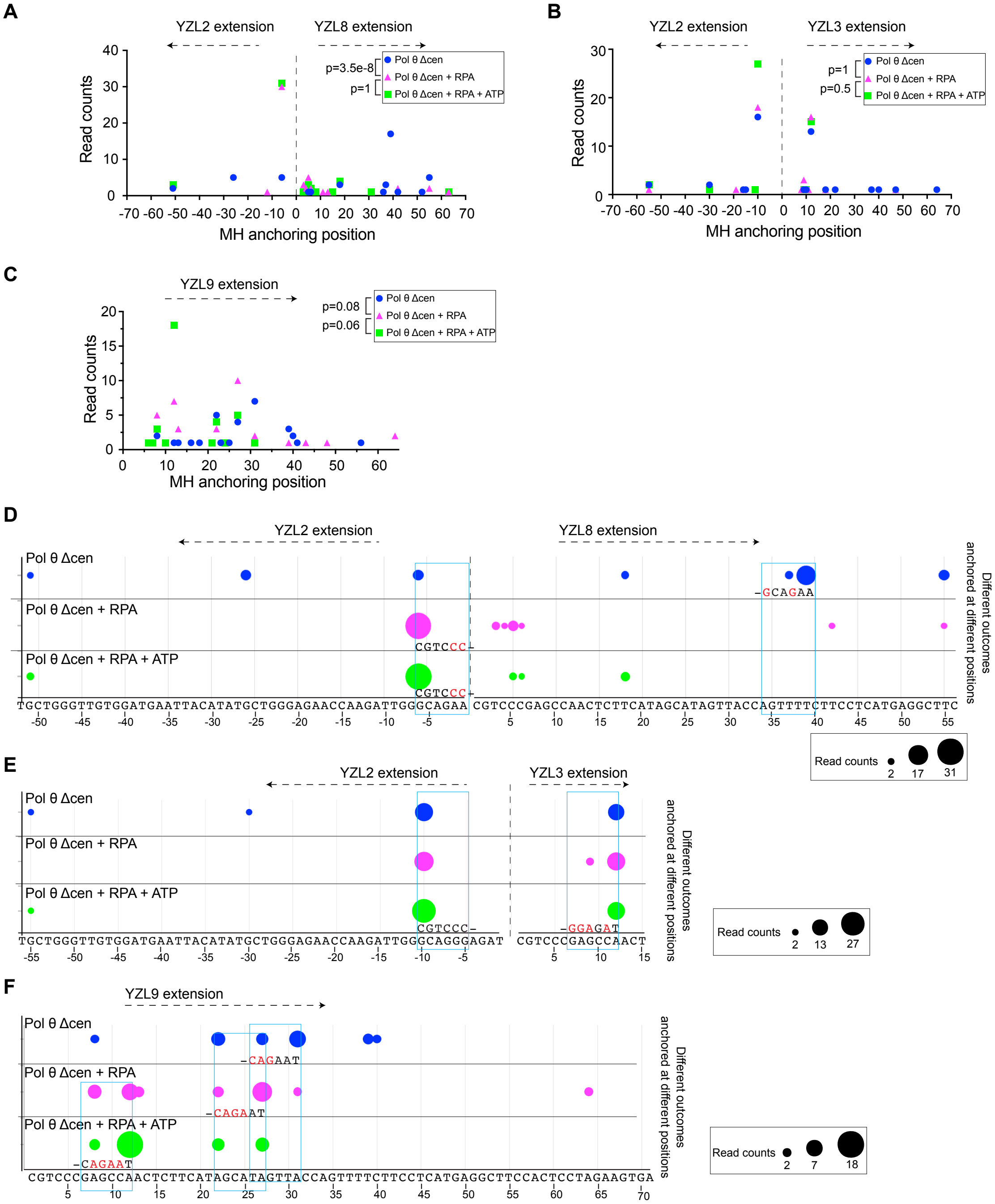
A
YZL2 extension
YZL8 extension
Read counts
MH anchoring position
p=3.5e-8
p=1
Pol θ Δcen
Pol θ Δcen + RPA
Pol θ Δcen + RPA + ATP
B
YZL2 extension
YZL3 extension
Read counts
MH anchoring position
p=1
p=0.5
Pol θ Δcen
Pol θ Δcen + RPA
Pol θ Δcen + RPA + ATP
C
YZL9 extension
Read counts
MH anchoring position
p=0.08
p=0.06
Pol θ Δcen
Pol θ Δcen + RPA
Pol θ Δcen + RPA + ATP
D
YZL2 extension
YZL8 extension
Pol θ Δcen
Pol θ Δcen + RPA
Pol θ Δcen + RPA + ATP
-GCAGAA
CGTCCC-
CGTCCC-
TGCTGGGTTGTGGATGAATTACATATGCTGGGAGAACCAAGATTGGGCAGAA CGTCCCGAGCCAACTCTTCATAGCATAGTTACCAGTTTTCTTCCTCATGAGGCTTC
Different outcomes anchored at different positions
Read counts 2 17 31
E
YZL2 extension
YZL3 extension
Pol θ Δcen
Pol θ Δcen + RPA
Pol θ Δcen + RPA + ATP
CGTCCC-
-GGAGAT
TGCTGGGTTGTGGATGAATTACATATGCTGGGAGAACCAAGATTGGGCAGGGAGAT CGTCCCGAGCCAACT
Different outcomes anchored at different positions
Read counts 2 13 27
F
YZL9 extension
Pol θ Δcen
Pol θ Δcen + RPA
Pol θ Δcen + RPA + ATP
-CAGAAT
-CAGAAT
-CAGAAT
CGTCCCGAGCCAACTCTTCATAGCATAGTTACCAGTTTTCTTCCTCATGAGGCTTCCACTCCTAGAAGTGA
Different outcomes anchored at different positions
Read counts 2 7 18

Figure S6. MH anchoring information and major junction position during TMEJ in Fig S5B.

(A-C) Summary of MH anchoring positions and read counts of TMEJ products in Fig S5B. The statistical significance is labeled with the p value derived from Kolmogorov-Smirnov test (KS-test).

(D-F) Major MH anchoring positions for the data in panels A-C. A circle marks each anchoring position of the primer ssDNA 3' end on the template ssDNA, indicating the junction where two ssDNAs are joined by Pol θ. Blue square indicates the major MH. The template ssDNA sequence is shown at the bottom, numbered from the 3' end. The size of the circle indicates the read count for each outcome. The colored circles indicate different reactions with Pol θ Δcen, with or without RPA, and ATP. The most frequent MH primer sequence is labeled under the corresponding circle. In the MH, bases in black are matched and bases in red are mismatched. Outcomes with only one read count were filtered out.

Fig S7

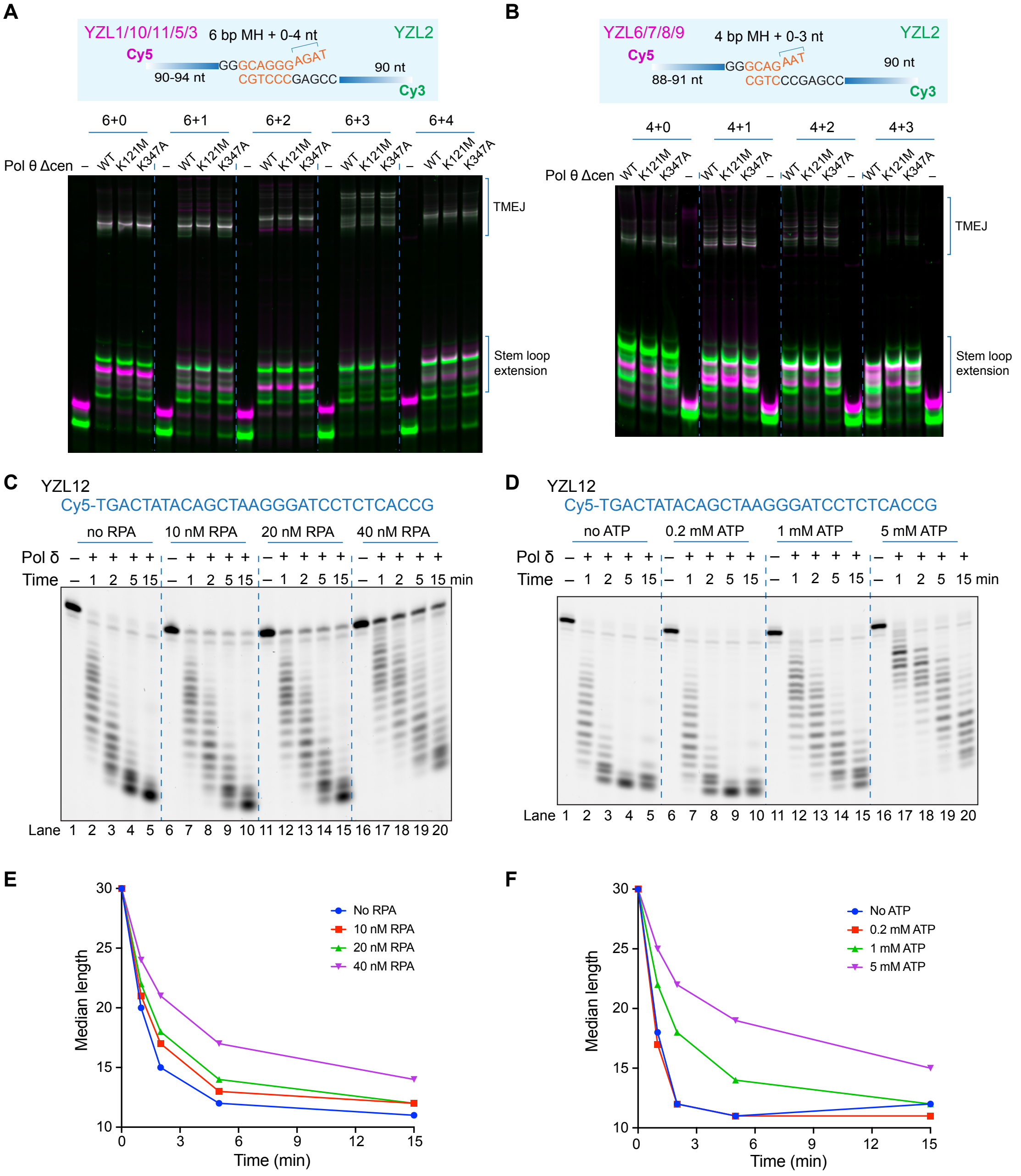

A
YZL1/10/11/5/3
6 bp MH + 0-4 nt
YZL2
Cy5
GGGCAGGGAGAT
90-94 nt
CGTCCCGAGCC
90 nt
Cy3
6+0
6+1
6+2
6+3
6+4
Pol θ Δcen
WT
K121M
K347A
TMEJ
Stem loop extension
B
YZL6/7/8/9
4 bp MH + 0-3 nt
YZL2
Cy5
GGGCAGAAT
88-91 nt
CGTCCCGAGCC
90 nt
Cy3
4+0
4+1
4+2
4+3
C
YZL12
Cy5-TGACTATACAGCTAAGGGATCCTCTCACCG
no RPA
10 nM RPA
20 nM RPA
40 nM RPA
Pol δ
Time
1 2 5 15 min
Lane 1 2 3 4 5 6 7 8 9 10 11 12 13 14 15 16 17 18 19 20
D
YZL12
Cy5-TGACTATACAGCTAAGGGATCCTCTCACCG
no ATP
0.2 mM ATP
1 mM ATP
5 mM ATP
E
Median length
Time (min)
No RPA
10 nM RPA
20 nM RPA
40 nM RPA
F
No ATP
0.2 mM ATP
1 mM ATP
5 mM ATP

Figure S7. Action of Pol θ Δcen variants and Pol δ on different DNA substrates.

(A) TMEJ reaction with Pol θ Δcen, K121M or K347A, and a series of ssDNA substrates (YZL1/10/11/5/3 and YZL2) which contain a designed 6 bp MH followed by 0 - 4 unpaired bases. A mixture of 25 nM each of the two indicated ssDNAs was incubated with 100 mU Pol θ Δcen, K121M and K347A in 15 µL reaction buffer at 37 ^{o}C for 15 min. Reaction mixtures were separated by electrophoresis on a native 10% polyacrylamide gel.

(B) TMEJ reaction with Pol θ Δcen, K121M or K347A, and series of ssDNA substrates (YZL6/7/8/9 and YZL2) which contains designed 4 bp MH followed by 0 - 3 unpaired bases. A mixture of 25 nM each of the two indicated ssDNAs was incubated with 100 mU Pol θ Δcen, K121M and K347A in 15 µL reaction buffer at 37 ^{o}C for 15 min. Reaction mixtures were separated by electrophoresis on a native 10% polyacrylamide gel.

(C) Effect of RPA on Pol δ exonuclease activity with 30-mer ssDNA. 20 nM YZL12 was pre-incubated with 0, 10, 20 or 40 nM RPA at 37 ^{o}C for 10 min and then incubated with 20 nM Pol δ in reaction buffer at 37 ^{o}C for the indicated times. Reaction mixtures were separated by electrophoresis on a denaturing 15% polyacrylamide gel.

(D) Effect of ATP on Pol δ exonuclease activity with 30-mer ssDNA. 20 nM YZL12 was pre-incubated with 5, 5.2, 6 or 10 mM $MgCl_2$ and then incubated with 20 nM Pol δ and 0, 0.2, 1, or 5 mM ATP in reaction buffer at 37 ^{o}C for the indicated times. Reaction mixtures were separated by electrophoresis on a denaturing 15% polyacrylamide gel.

(E) Quantification of the median length of Pol δ digested fragments at different time points in (C). The intensity of each band was measured by ImageJ. The median length is the point where half of the lane intensity was above and half below this point.

(F) Quantification of the median length of Pol δ digested fragments at different time points in (D). The intensity of each band was measured by ImageJ. The median length is the point where half of the lane intensity was above and half below this point.

Fig S8

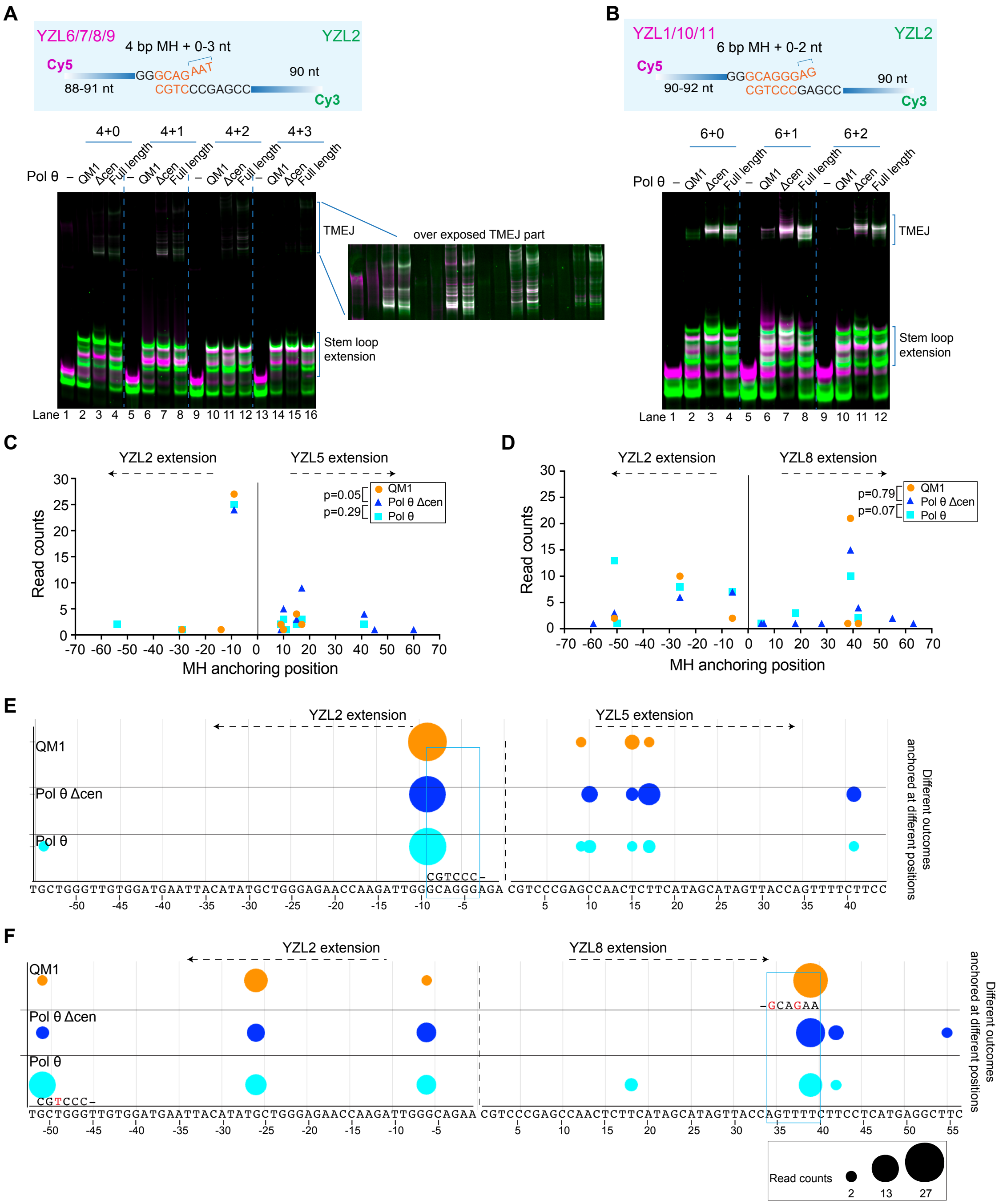
A
YZL6/7/8/9
YZL2
4 bp MH + 0-3 nt
Cy5
88-91 nt
90 nt
Cy3
4+0
4+1
4+2
4+3
Pol θ
TMEJ
over exposed TMEJ part
Stem loop extension
Lane 1 2 3 4 5 6 7 8 9 10 11 12 13 14 15 16
B
YZL1/10/11
YZL2
6 bp MH + 0-2 nt
Cy5
90-92 nt
90 nt
Cy3
6+0
6+1
6+2
Pol θ
TMEJ
Stem loop extension
Lane 1 2 3 4 5 6 7 8 9 10 11 12
C
YZL2 extension
YZL5 extension
Read counts
MH anchoring position
p=0.05
p=0.29
QM1
Pol θ Δcen
Pol θ
D
YZL2 extension
YZL8 extension
Read counts
MH anchoring position
p=0.79
p=0.07
QM1
Pol θ Δcen
Pol θ
E
YZL2 extension
YZL5 extension
QM1
Pol θ Δcen
Pol θ
Different outcomes anchored at different positions
F
YZL2 extension
YZL8 extension
QM1
Pol θ Δcen
Pol θ
Different outcomes anchored at different positions
Read counts
2
13
27

Figure S8. The strand capture ability of Pol θ HLD is important for TMEJ.

(A) TMEJ reaction with QM1, Pol θ Δcen or full-length Pol θ and ssDNA substrates which contains a designed 4 bp MH followed by 0 - 3 unpaired bases. 25 nM YZL6/7/8/9 and 25 nM YZL2 were incubated with 30 mU QM1, Pol θ Δcen or full-length Pol θ in 15 μL reaction buffer at 37 °C for 20 min. Reaction mixtures were separated by electrophoresis on a native 10% polyacrylamide gel.

(B) TMEJ reaction with QM1, Pol θ Δcen or full-length Pol θ and ssDNA substrates which contains a designed 6 bp MH followed by 0 - 2 unpaired bases. 25 nM YZL1/10/11 and 25 nM YZL2 were incubated with 30 mU QM1, Pol θ Δcen or full-length Pol θ in 15 μL reaction buffer at 37 °C for 20 min. Reaction mixtures were separated by electrophoresis on a native 10% polyacrylamide gel.

(C) Summary of MH anchoring positions and read counts of YZL5/YZL2 TMEJ products in Fig 4G. The statistical significance is labeled with the p value derived from Kolmogorov-Smirnov test (KS-test).

(D) Summary of MH anchoring positions and read counts of YZL8/YZL2 TMEJ products in (A). The statistical significance is labeled with the p value derived from Kolmogorov-Smirnov test (KS-test).

(E-F) Major MH anchoring positions for the data in (C-D). A circle marks each anchoring position of the primer ssDNA 3' end on the template ssDNA, indicating the junction where two ssDNAs are joined by Pol θ. Blue square indicates the major MH. The template ssDNA sequence is shown at the bottom, numbered from the 3' end. The size of the circle indicates the read count for each outcome. The colored circles indicate different reactions with QM1, Pol θ Δcen, or full-length Pol θ. The most frequent MH primer sequence is labeled under the corresponding circle. In the MH, bases in black are matched and bases in red are mismatched. Outcomes with only one read count were filtered out.

Fig S9

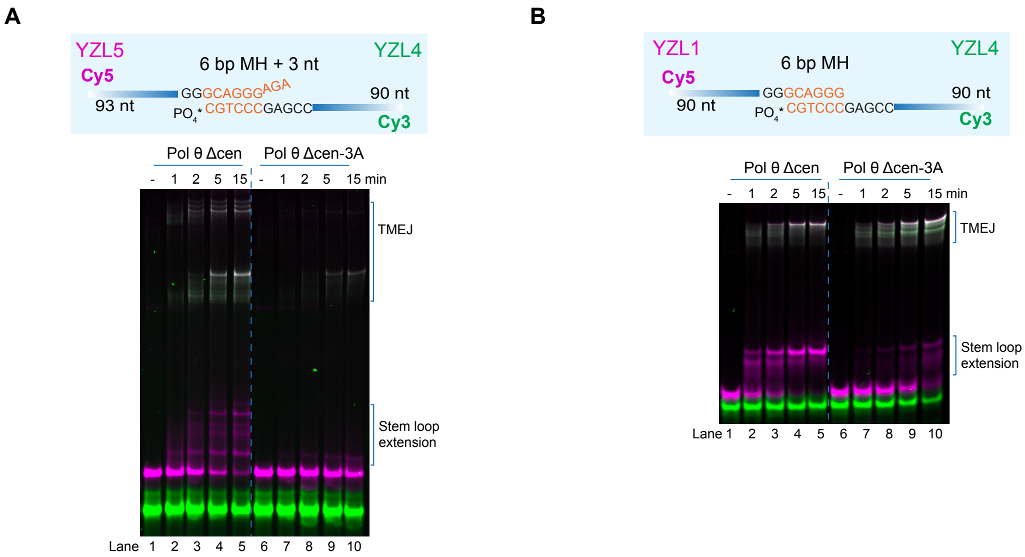

Figure S9. The primer-grasp amino acids of Pol θ POL are important for TMEJ.

(A) TMEJ reaction with Pol θ Δcen or its variant 3A and ssDNA substrate. 25 nM YZL5 and 25 nM YZL4 were incubated with 80 mU Pol θ Δcen or its variant 3A in 20 μL reaction buffer at 37 °C for indicated time points. Reaction mixtures were separated by electrophoresis on a native 10% polyacrylamide gel.

(B) TMEJ reaction with Pol θ Δcen or its variant 3A and ssDNA substrate. 25 nM YZL1 and 25 nM YZL4 were incubated with 80 mU Pol θ Δcen or its variant 3A, and 100 μM dNTPs in 20 μL reaction buffer at 37 °C for indicated time points. Reaction mixtures were separated by electrophoresis on a native 10% polyacrylamide gel.

Fig S10

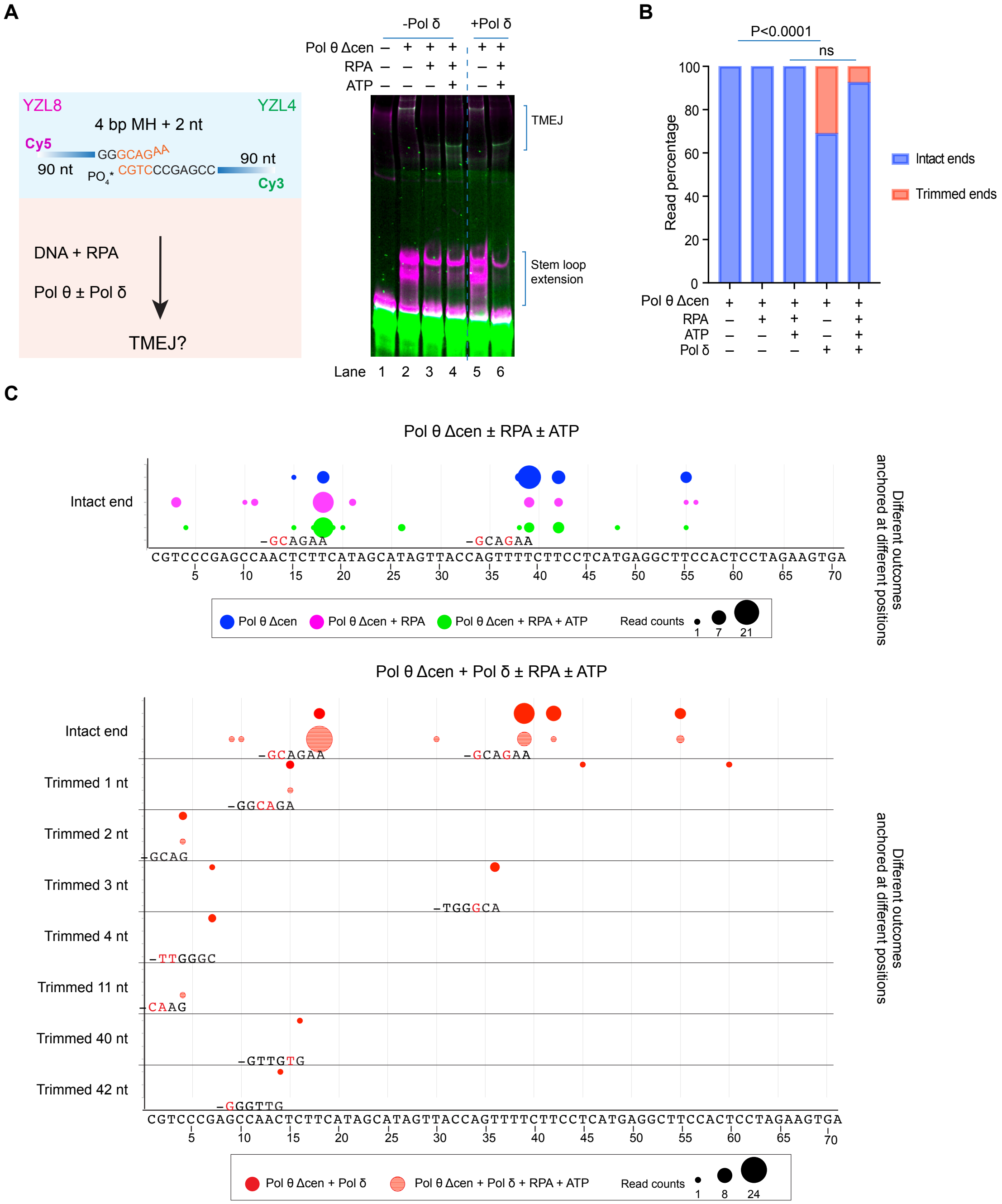

A
YZL8
YZL4
4 bp MH + 2 nt
Cy5
GGGCAGAA
90 nt
90 nt
PO4
CGTCCCGAGCC
Cy3
DNA + RPA
Pol θ ± Pol δ
TMEJ?
-Pol δ
+Pol δ
Pol θ Δcen
RPA
ATP
TMEJ
Stem loop extension
Lane 1 2 3 4 5 6
B
P<0.0001
ns
Read percentage
Intact ends
Trimmed ends
Pol θ Δcen
RPA
ATP
Pol δ
C
Pol θ Δcen ± RPA ± ATP
Intact end
-GCAGAA
CGTCCCGAGCCAACTCTTCATAGCATAGTTACCAGTTTTCTTCCTCATGAGGCTTCCACTCCTAGAAGTGA
Pol θ Δcen
Pol θ Δcen + RPA
Pol θ Δcen + RPA + ATP
Read counts
1
7
21
Different outcomes anchored at different positions
Pol θ Δcen + Pol δ ± RPA ± ATP
Intact end
Trimmed 1 nt
Trimmed 2 nt
Trimmed 3 nt
Trimmed 4 nt
Trimmed 11 nt
Trimmed 40 nt
Trimmed 42 nt
-GCAGAA
-GGCAGA
-GCAG
-TGGGCA
-TTGGGC
-CAAG
-GTTGTG
-GGGTTG
CGTCCCGAGCCAACTCTTCATAGCATAGTTACCAGTTTTCTTCCTCATGAGGCTTCCACTCCTAGAAGTGA
Pol θ Δcen + Pol δ
Pol θ Δcen + Pol δ + RPA + ATP
Read counts
1
8
24
Different outcomes anchored at different positions

Figure S10. Functional TMEJ reconstitution.

(A) TMEJ reconstitution with Pol θ Δcen, Pol δ, RPA, ATP and DNA substrate. 25 nM YZL8 and 25 nM YZL4 were pre-incubated with 300 nM RPA and 6 mM $MgCl_2$ at 37 °C for 10 min and then incubated with different combinations of 100 nM Pol θ Δcen, 100 nM Pol δ, 1 mM ATP, and 50 μM dNTPs in reaction buffer at 37 °C for 15 min. Reaction mixtures were separated by electrophoresis on a native 10% polyacrylamide gel.

(B) Read percentage of TMEJ outcomes arising from intact or trimmed ends of YZL8 from samples in panel A (lanes 2-6). The statistical significance is labeled with the p value derived from two-sided Fisher's exact test. ns indicates no significant difference.

(C) MH anchoring positions for the data in panel B. A circle marks each anchoring position of the YZL8 3' end on the YZL4 template, indicating the junction where two ssDNAs are joined by Pol θ. The YZL4 template sequence is shown at the bottom, numbered from the 3' end. The size of the circle indicates the read count for each outcome. The colored circles indicate different reactions with Pol θ Δcen, with or without RPA, ATP, and Pol δ. Outcomes are categorized based on whether they resulted from an intact 3' end or from a trimmed YZL8 3' end. The most frequent MH primer sequence is labeled under the corresponding circle. In the MH, bases in black are matched and bases in red are mismatched.

Fig S11

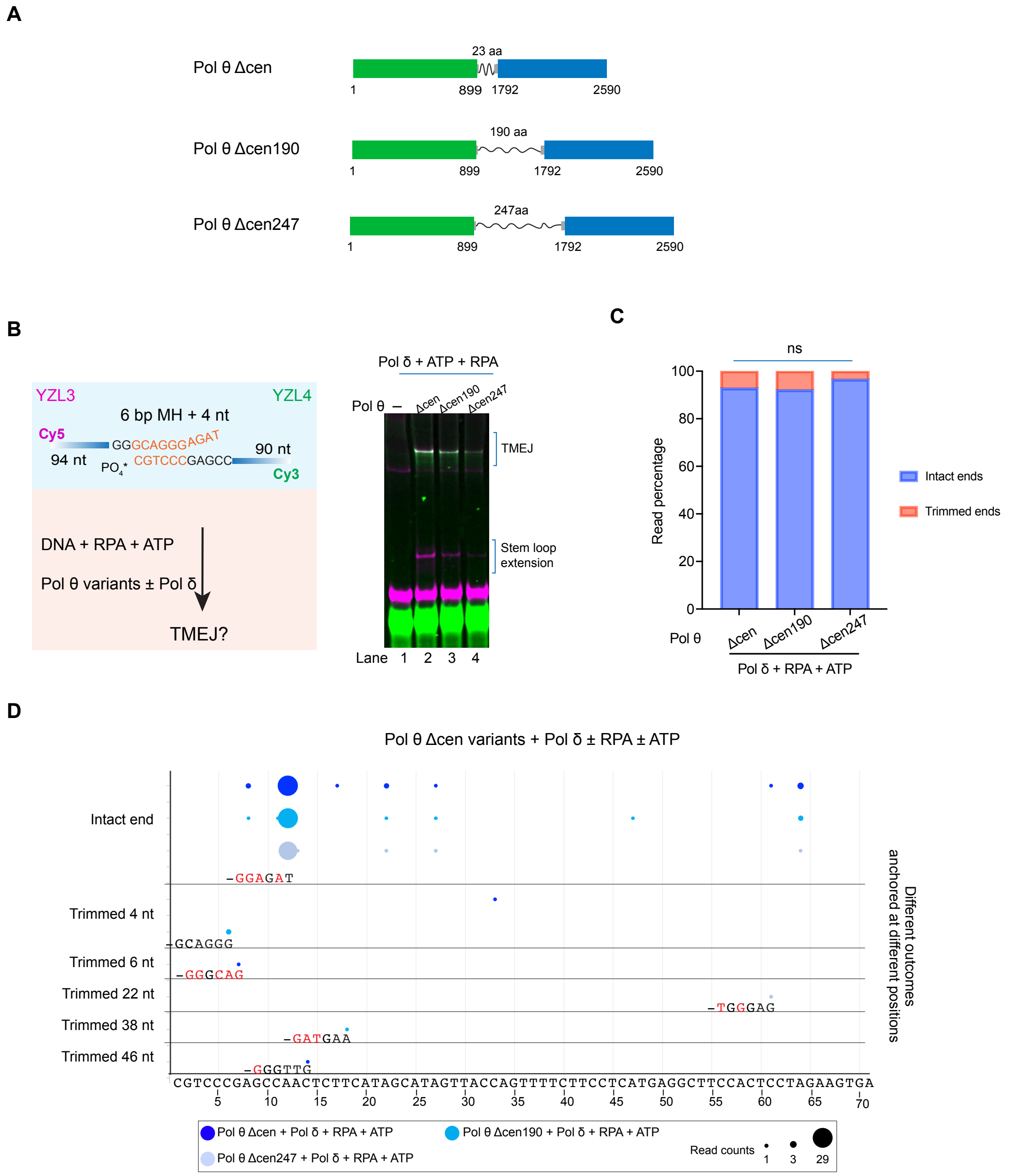
A
Pol θ Δcen
23 aa
1
899
1792
2590
Pol θ Δcen190
190 aa
Pol θ Δcen247
247aa
B
YZL3
YZL4
6 bp MH + 4 nt
Cy5
94 nt
90 nt
Cy3
DNA + RPA + ATP
Pol θ variants ± Pol δ
TMEJ?
Pol δ + ATP + RPA
Pol θ
Δcen
Δcen190
Δcen247
TMEJ
Stem loop extension
Lane 1 2 3 4
C
ns
Read percentage
Intact ends
Trimmed ends
Pol δ + RPA + ATP
D
Pol θ Δcen variants + Pol δ ± RPA ± ATP
Intact end
Trimmed 4 nt
Trimmed 6 nt
Trimmed 22 nt
Trimmed 38 nt
Trimmed 46 nt
Different outcomes anchored at different positions
CGTCCCGAGCCAACTCTTCATAGCATAGTTACCAGTTTTCTTCCTCATGAGGCTTCCACTCCTAGAAGTGA
Pol θ Δcen + Pol δ + RPA + ATP
Pol θ Δcen190 + Pol δ + RPA + ATP
Pol θ Δcen247 + Pol δ + RPA + ATP
Read counts
1
3
29

Figure S11. Functional TMEJ reconstitution with Pol θ Δcen variants.

(A) Diagram of Pol θ Δcen construct and its two variants with different length of central domain.

(B) TMEJ reconstitution with Pol θ Δcen or its variants, Pol δ, RPA, ATP and DNA substrate. 25 nM YZL3 and 25 nM YZL4 were pre-incubated with 300 nM RPA and 6 mM $MgCl_2$ at 37 ^oC for 10 min and then incubated with 100 nM Pol θ Δcen or its variants, 100 nM Pol δ, 1 mM ATP, and 50 μM dNTPs in reaction buffer at 37 ^oC for 15 min. Reaction mixtures were separated by electrophoresis on a native 10% polyacrylamide gel.

(C) Read percentage of TMEJ outcomes arising from intact or trimmed ends of YZL3 from samples in panel B (lanes 2-4). The statistical significance derived from two-sided Fisher's exact test shows no significant difference (ns) among them.

(D) MH anchoring positions for the data in panel C. A circle marks each anchoring position of the YZL3 3' end on the YZL4 template, indicating the junction where two ssDNAs are joined by Pol θ. The YZL4 template sequence is shown at the bottom, numbered from the 3' end. The size of the circle indicates the read count for each outcome. The colored circles indicate different reactions with Pol θ Δcen (or its variants), RPA, ATP, and Pol δ. Outcomes are categorized based on whether they resulted from an intact 3' end or from a trimmed YZL3 3' end. The most frequent MH primer sequence is labeled under the corresponding circle. In the MH, bases in black are matched and bases in red are mismatched.

Fig S12

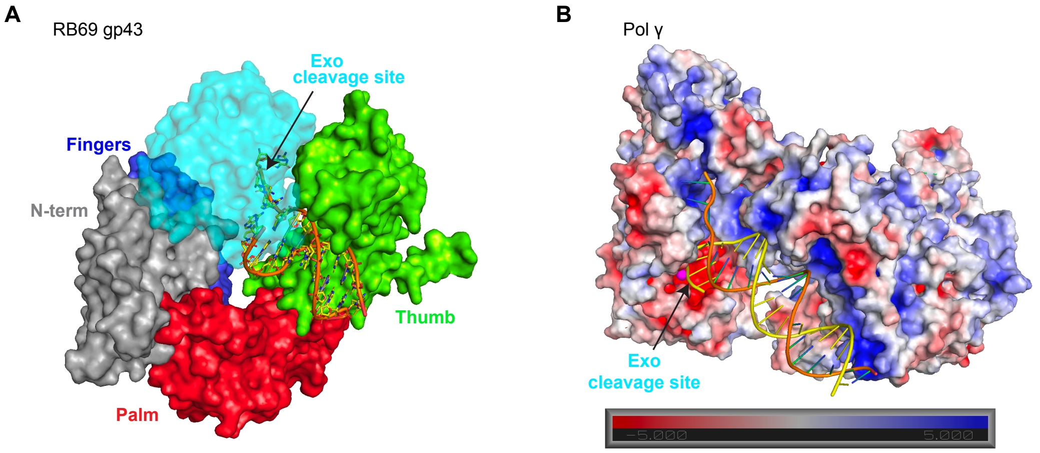

Figure S12. 3' single-stranded DNA is fully encompassed in editing sites of DNA polymerases.

(A) View of DNA in the editing site of a family B Pol δ homolog (RB69 gp43). In this structure (PDB ID code 1CLQ (1)) with primer template DNA in editing mode, about 10 nucleotides of the edited strand are contacted by the polymerase. Four terminal 3' nucleotides are fully within the channel of the exonuclease domain. Domains are color coded as follows: N-terminal (gray), exonuclease (cyan), palm (red), fingers (blue) and thumb (green).

(B) Stick view of DNA in the editing site of a family A DNA polymerase, human Pol γ. In this structure (PDB ID code 8D42 (2)), > 10 bases of the edited strand (yellow) are contacted by the enzyme, with 4 nt within the editing channel. In this view the surface displays the electrostatic potential map (blue, positive; red, negative). Molecular figures made with PyMOL (3).

Table S1. Oligonucleotide sequences.

Stock Oligo Name	Oligo Names	5'-end	5'-Sequence-3'	3' end	Length
Cy5-90-6MH-1	YZL1	Cy5	TGACTATACAGCTAAGGGATCCTCTCACCGAGCGTATCTGCTGGGTTGTGG ATGAATTACATATGCTGGGAGAACCAAGATTGGGCAGGG	3'-OH	90
Cy3-90-6MH-7	YZL2	Cy3	AGTCTGAGCTCGGTGTGAGAGTGAAGATCCTCACCTTCGGAGTACTCCTTC TTTTGACCATTGATACGATACTTCTCAACCGAGCCCTGC	3'-OH	90
Oligo 6N4B	YZL3	Cy5	TGACTATACAGCTAAGGGATCCTCTCACCGAGCGTATCTGCTGGGTTGTGG ATGAATTACATATGCTGGGAGAACCAAGATTGGGCAGGGAGAT	3'-OH	94
oligo10	YZL4	Cy3	AGTCTGAGCTCGGTGTGAGAGTGAAGATCCTCACCTTCGGAGTACTCCTTC TTTTGACCATTGATACGATACTTCTCAACCGAGCCCTGC	3'-PO4	90
Oligo 5N3B	YZL5	Cy5	TGACTATACAGCTAAGGGATCCTCTCACCGAGCGTATCTGCTGGGTTGTGG ATGAATTACATATGCTGGGAGAACCAAGATTGGGCAGGGAGA	3'-OH	93
oligo 0N0M	YZL6	Cy5	TGACTATACAGCTAAGGGATCCTCTCACCGAGCGTATCTGCTGGGTTGTGG ATGAATTACATATGCTGGGAGAACCAAGATTGGGCAG	3'-OH	88
Oligo 1N1M	YZL7	Cy5	TGACTATACAGCTAAGGGATCCTCTCACCGAGCGTATCTGCTGGGTTGTGG ATGAATTACATATGCTGGGAGAACCAAGATTGGGCAGA	3'-OH	89
Oligo 2N2M	YZL8	Cy5	TGACTATACAGCTAAGGGATCCTCTCACCGAGCGTATCTGCTGGGTTGTGG ATGAATTACATATGCTGGGAGAACCAAGATTGGGCAGAA	3'-OH	90
Oligo 3N3M	YZL9	Cy5	TGACTATACAGCTAAGGGATCCTCTCACCGAGCGTATCTGCTGGGTTGTGG ATGAATTACATATGCTGGGAGAACCAAGATTGGGCAGAAT	3'-OH	91
Oligo 3N1B	YZL10	Cy5	TGACTATACAGCTAAGGGATCCTCTCACCGAGCGTATCTGCTGGGTTGTGG ATGAATTACATATGCTGGGAGAACCAAGATTGGGCAGGGA	3'-OH	91
Oligo 4N2B	YZL11	Cy5	TGACTATACAGCTAAGGGATCCTCTCACCGAGCGTATCTGCTGGGTTGTGG ATGAATTACATATGCTGGGAGAACCAAGATTGGGCAGGGAG	3'-OH	92
oilgo1-30	YZL12	Cy5	TGACTATACAGCTAAGGGATCCTCTCACCG	3'-OH	30
F2	F	None	GCCTGCAGGTCGACTTGACTATACAGCTAAGGGATCC	3'-OH	37
R1/2	R	None	AAACGACGGCCAGTGAGTCTGAGCTCGGTGTGAG	3'-OH	34
Forward-seq	F-Seq	None	CACTTTATGCTTCCGGCTCG	3'-OH	20
Cy5-16 bottom	P/T	Cy5	TGAGTGGTACTGTGAG	3'-OH	16
30-16 bottom		None	CTCGTCAGCATCTTCTCACAGTACCACTCA	3'-OH	30
ACCA-14e	YZL0	Cy5	ACCAGCGGCTGTCATAGC	3'-OH	18
Oligo 30F	30F	Cy5	ATATGCTGGGAGAACCAAGATTGGGCAGGG	3'-OH	30
Oligo 7-30	30R	Cy3	TTGATACGATACTTCTCAACCGAGCCCTGC	3'-OH	30
Oligo 60+0M	60F	Cy5	CGAGCGTATCTGCTGGGTTGTGGATGAATTACATATGCTGGGAGAACCAAG ATTGGGCAG	3'-OH	60
Oligo 7-60	60R	Cy3	ACCTTCGGAGTACTCCTTCTTTTGACCATTGATACGATACTTCTCAACCGAG CCCTGCCC	3'-OH	60

SI References

1. M. Hogg, S. S. Wallace, S. Doublié, Crystallographic snapshots of a replicative DNA polymerase encountering an abasic site. *EMBO J* **23**, 1483-1493 (2004).
2. J. Park, G. K. Herrmann, P. G. Mitchell, M. B. Sherman, Y. W. Yin, Polγ coordinates DNA synthesis and proofreading to ensure mitochondrial genome integrity. *Nat Struct Mol Biol* **30**, 812-823 (2023).
3. Schrödinger, LLC, The PyMOL Molecular Graphics System, Version 3.1.3. (2015).
